# Thrombospondin 1 promotes an aggressive phenotype through epithelial-to-mesenchymal transition in human melanoma

**DOI:** 10.18632/oncotarget.2164

**Published:** 2014-07-08

**Authors:** Aparna Jayachandran, Matthew Anaka, Prashanth Prithviraj, Christopher Hudson, Sonja J McKeown, Pu-Han Lo, Laura J Vella, Colin R Goding, Jonathan Cebon, Andreas Behren

**Affiliations:** ^1^ Ludwig Institute for Cancer Research, Melbourne-Austin Branch, Cancer Immunobiology Laboratory, Heidelberg, VIC 3084, Australia; ^2^ Department of Medicine, University of Melbourne, Victoria, 3010, Australia; ^3^ Department of Anatomy and Neuroscience, University of Melbourne, Victoria, 3010, Australia; ^4^ Ludwig Institute for Cancer Research, University of Oxford, Oxford, OX3 7DQ, UK

**Keywords:** Thrombospondin 1, melanoma, epithelial-to-mesenchymal transition, chick embryo, invasion, drug resistance

## Abstract

Epithelial-to-mesenchymal transition (EMT), in which epithelial cells loose their polarity and become motile mesenchymal cells, is a determinant of melanoma metastasis. We compared gene expression signatures of mesenchymal-like melanoma cells with those of epithelial-like melanoma cells, and identified Thrombospondin 1 (*THBS1*) as highly up-regulated in the mesenchymal phenotype. This study investigated whether THBS1, a major physiological activator of transforming growth factor (TGF)-beta, is involved in melanoma EMT-like process. We sought to examine expression patterns in distinct melanoma phenotypes including invasive, de-differentiated, label-retaining and drug resistant populations that are putatively associated with an EMT-like process.

Here we show that THBS1 expression and secretion was elevated in melanoma cells exhibiting invasive, drug resistant, label retaining and mesenchymal phenotypes and correlated with reduced expression of genes involved in pigmentation. Elevated THBS1 levels were detected in Vemurafenib resistant melanoma cells and inhibition of *THBS1* led to significantly reduced chemoresistance in melanoma cells. Notably, siRNA-mediated silencing of *THBS1* and neutralizing antibody to THBS1 reduced invasion in mesenchymal-like melanoma cells, while ectopic *THBS1* expression in epithelial-like melanoma cells enhanced invasion. Furthermore, the loss of *THBS1* inhibited *in vivo* motility of melanoma cells within the embryonic chicken neural tube. In addition, we found aberrant THBS1 protein expression in metastatic melanoma tumor biopsies. These results implicate a role for THBS1 in EMT, and hence THBS1 may serve as a novel target for strategies aimed at the treatment of melanoma invasion and drug resistance.

## INTRODUCTION

Melanoma is a frequently fatal malignancy of the neural crest-derived melanocytes, the pigment producing cells in skin, uveal tract and mucosal membranes. The cause of death from melanoma is metastasis [1]. While new treatments targeting specific melanoma driver mutations such as BRAF V600E have a significant impact, the benefit of treatment is often short lived and the disease becomes rapidly fatal once resistance develops [2, 3]. Accumulating evidence indicates that the acquisition of invasive and metastatic characteristics by melanoma cells involves the reactivation of a developmental epithelial-to-mesenchymal transition (EMT)-like program [4-6]. The complex mechanisms involved in this process in melanoma remain largely unknown.

EMT describes a reprogramming of epithelial cells that leads to the loss of epithelial characteristics, notably polarity and cell adhesion, and the acquisition of a mesenchymal phenotype with increased invasive abilities. It occurs during normal development as part of processes such as gastrulation and neural crest cell migration [7]. During cancer progression, this phenotype is associated with tumor invasion, metastatic dissemination, and acquisition of resistance to drug treatment [8, 9]. Cadherin switching is a hallmark of EMT, leading to the down-regulation and replacement of the cell surface adhesion molecule E-cadherin by N-cadherin, enabling motility [10-12].

In various cancers, EMT leads to the generation of cancer cells possessing stem cell attributes of tumor-initiation and resistance to chemotherapy [8]. In melanoma, a sub-population of slow-cycling cells (defined by label-retention) which exhibit efficient tumor-initiating capacity, have properties associated with stemness, and are resistant to various anticancer drugs has been identified [13].

Thrombospondins comprise a family of homologous proteins that regulate cellular phenotype and extracellular structure during tissue genesis and remodelling. Thrombospondin 1 (*THBS1*) was the first member to be identified, and has been shown to modulate tumor progression and metastasis [14, 15]. Whereas the role of *THBS1* in angiogenesis in melanoma is well documented, its role in tumor metastasis is only just emerging [16, 17]. *THBS1* has been identified as a major physiological activator of transforming growth factor (TGF)-beta, a potent elicitor of EMT [18, 19]. However, a role of *THBS1* in mediating EMT in melanoma remains to be elucidated. This study therefore aimed to reveal the functional roles of *THBS1* during melanoma progression by assessing *THBS1* expression and its effects on the functional characteristics of melanoma cells, particularly those associated with mesenchymal transformation.

## RESULTS

### Melanoma cells exhibiting a mesenchymal phenotype express high levels of Thrombospondin 1 associated with TGF-beta signaling

**Figure 1:**
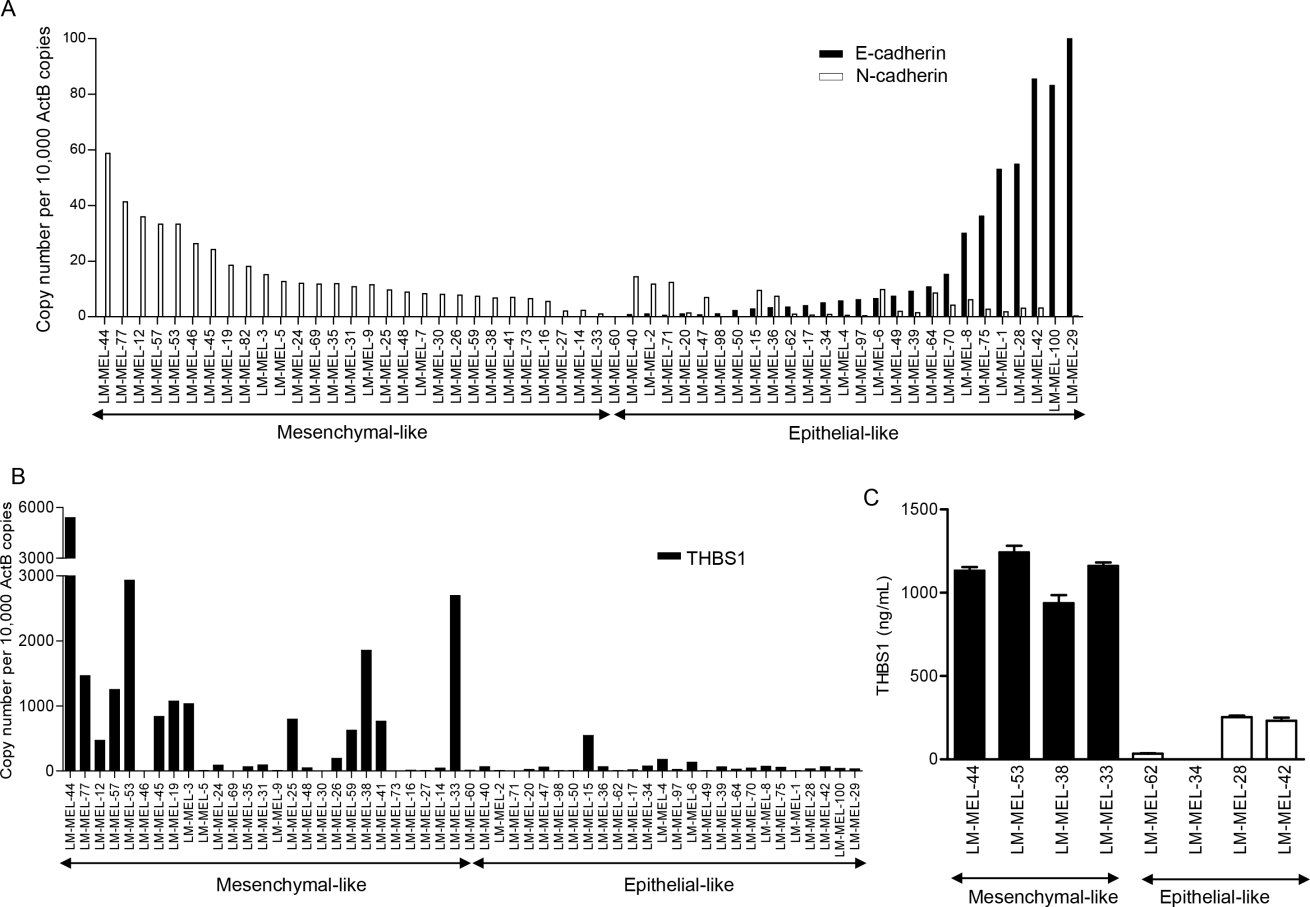
Classification of a panel of melanoma cells lines based on gene expression qRT-PCR analysis revealed expression of (**A**) E- and N-cadherins and (**B**) *THBS1* in a panel of 54 melanoma cell lines. (**C**) Media from 4 mesenchymal- and 4 epithelial-like melanoma cells were collected and subjected to THBS1 ELISA. ANOVA analysis of the two sets of cell lines was significant (p<0.005).

At the molecular level, EMT in melanoma cells is characterized by a series of coordinated changes including down-regulation of the adherens junction molecule E-cadherin and upregulation of N-cadherin [5, 20]. These changes in EMT markers are often associated with functional change toward an invasive phenotype [21]. We evaluated the expression of classical EMT genes, E- and N-cadherins using quantitative real-time RT-PCR (qRT-PCR) in a panel of 54 human melanoma cell lines that were derived from resected melanoma metastases [22]. Expression patterns of these two molecules in the cell lines varied from high N-cadherin with no E-cadherin expression, high E-cadherin with no or low N-cadherin, to intermediate levels of both (Figure 1A).

We divided the lines into those expressing E-cadherin and those lacking E-cadherin expression, and their gene expression patterns were compared using previously generated whole genome microarray expression profiling data [22]. 634 probes representing 552 genes were differentially expressed between the two classes of cell lines (Supplementary Table S1). As expected E-cadherin expression was higher in the lines identified as E-cadherin expressing by qRT-PCR (13.5 fold), and N-cadherin expression was higher in the lines lacking E-cadherin expression by qRT-PCR. A principal components analysis based on the differential expression gene list largely segregated the two classes of cell lines, although with some overlap between lines with intermediate levels of E- and N-cadherin, perhaps representing a mixed phenotype (Supplementary Figure S1). A gene-set enrichment analysis (GSEA) of the cell lines revealed gene sets associated with TGF-beta signaling [23], cell migration [24, 25], ECM modulation [26] and EMT [27-29]. Significantly enriched gene sets can be found in Supplementary Table S2. Based on the GSEA results, and the evidence of opposing E- and N-cadherin expression, we therefore labelled the cell lines mesenchymal- and epithelial-like.

We chose to focus on Thrombospondin 1 (*THBS1*), which showed 19 fold higher expression in the mesenchymal-like cells. qRT-PCR confirmed *THBS1* mRNA expression levels were higher in mesenchymal-like cells (Figure 1B). A subset of high and low *THBS1* expressing cell lines, as determined by qRT-PCR, was subjected to solid-phase ELISA. This detected little or no THBS1 secretion in conditioned medium from epithelial-like cells, whereas mesenchymal-like cells secreted significant amounts of THBS1 into the medium (Figure 1C).

As THBS1 is a known activator of TGF-beta [18] and TGF-beta has a pivotal function in the progression of EMT [30, 31], we examined the level of TGF-beta secretion in a subset of high and low THBS1 secreting melanoma cell lines. THBS1 high cell lines secreted high TGF-beta1 in contrast to THBS1 low cell lines that secreted no TGF-beta1 into the medium (Supplementary Figure S2A). To extend these findings, we analyzed a cutaneous melanoma dataset available from The Cancer Genome Atlas (TCGA) (http://www.cbioportal.org) [32, 33]. Mutual exclusivity data from 376 melanoma patients revealed that both THBS1 and TGF-beta1 are co-expressed (Odds ratio =3.4, p=0.036, Fisher's exact test). TGF-beta1 treatment in two epithelial-like melanoma cell lines induced *THBS1* expression in a time-dependent manner (Supplementary Figure S2B). These data indicate that *THBS1* expression is associated with TGF-beta signaling in melanoma cells.

### Thrombospondin 1 expression negatively correlates with differentiation markers of melanoma

De-differentiation, which is characterized by the loss of expression of genes involved in pigmentation, is often associated with aggressive phenotype in melanoma [6, 33]. A key determinant of melanoma differentiation sub-population identity is conferred by the expression and activation of microphthalmia-associated transcription factor (*MITF*). In melanoma, the induction of MITF expression promotes expression of differentiation markers and the inhibition of invasion [35]. Differentiation markers MelanA (*MLANA*) (fold change: 37), Tyrosinase (*TYR)* (fold change: 27), and *MITF* (fold change: 10) were up-regulated in the epithelial-like cells examined. qRT-PCR analysis showed expression of melanoma differentiation markers *MLANA*, *TYR* and *MITF* in the panel of 54 melanoma cell lines. Regression analysis revealed an inverse correlation of *THBS1* expression with all three genes tested (p<0.005 in each case) (Supplementary Figure S3). This demonstrates the association between *THBS1* expression and a de-differentiated phenotype in melanoma.

### Thrombospondin 1 expression is enriched in label-retaining melanoma cells

**Figure 2:**
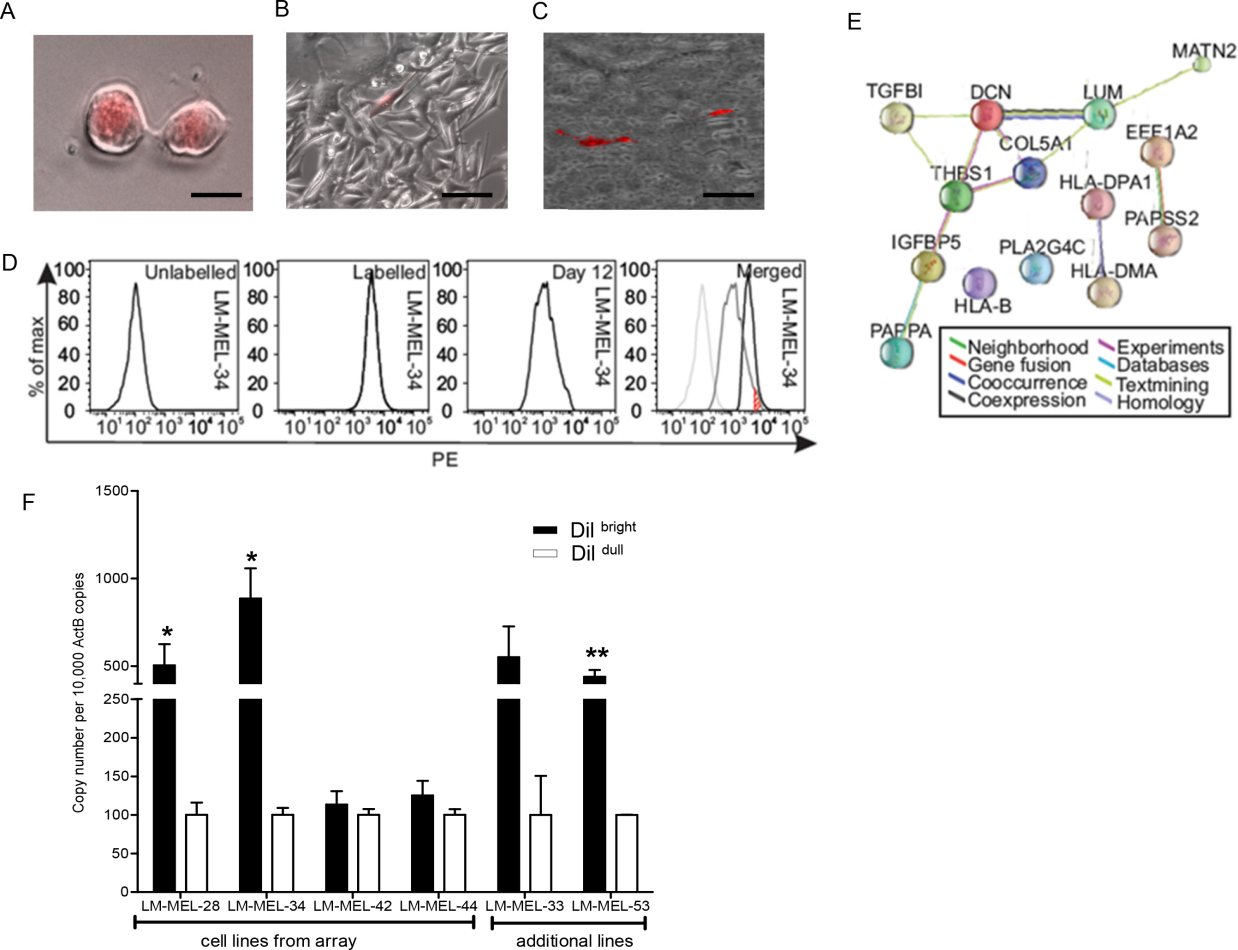
Expression of *THBS1* in LRC sub-population within melanoma cell lines Melanoma cell line, LM-MEL-34 labelled with CM-DiI was imaged after (**A**) 2 days (scale bar = 50μm) or (**B**) 14 days (scale bar = 100μm), respectively. (**C**) Melanoma cells from a patient with malignant melanoma were labelled with CM-Dil and injected subcutaneously into the flank of three animals. After three weeks, tumors were harvested and embedded in paraffin, cut into sections and examined by fluorescent microscopy (scale bar = 500μm). (**D**) Example of a flow profile of LM-MEL-34 cells unstained, 1 hour after CM-Dil labeling, 12 days after labeling, and merged histogram overlay (from left to right). The red striped area represents the population of cells referred to as LRC. LM-MEL−28, −34, −42 and −44 were sorted into Dil^bright^ and Dil^dull^ cells 14 days after labeling with CM-Dil. RNA was subjected to genome-wide gene expression analysis using Illumina HT12 arrays. (**E**) Upregulated genes were employed to generate an evidence-based protein-interaction network in STRING and demonstrated connections between several ECM-related molecules with higher expression in the Dil^bright^ cells. (**F**) Dil^bright^ and Dil^dull^ cells from the same 4 lines and 2 additional cell lines were separated and qPCR was performed to evaluate expression of *THBS1*. Values are mean +/− SEM of three experiments in triplicate (* p<0.05, ** p<0.005).

Others have described label retention as an assay for slow-cycling melanoma cells required for continuous tumor maintenance [13]. To assess whether *THBS1* expression is associated with label-retaining cells (LRC) in melanoma, we isolated these cells by using CM-Dil dye, a membrane labeling carbocyanine dye that was distributed equally between daughter cells with each cell division (Figure 2A). After culturing the labelled cells for two weeks, the majority of cells diluted the label to undetectable levels, while a sub-population of cells continued to be brightly labelled (Figure 2B). To ensure that these LRC were not an *in vitro* artifact of the culture conditions employed, we labelled melanoma cells freshly isolated from the ascites of a patient with advanced metastatic melanoma and immediately injected them subcutaneously into the flank of NOD/SCID mice. After three weeks of tumor growth, a small percentage of LRC could be visualized in a paraffin-embedded xenograft specimen (Figure 2C).

The ‘label-retaining’ phenotype was present in all melanoma cell lines tested (n=10) *in vitro* and could be visualized by flow cytometry (Figure 2D). Fourteen days after labeling, a small population of cells showed signal intensities close to those measured directly after labeling (Supplementary Figure S4). LRC were therefore FACS-sorted from 4 cell lines based on dye-retention (Dil^bright^) and subjected to a whole genome expression analysis. Genes significantly up- or down-regulated are listed in Supplementary Table S3. *THBS1* was significantly up-regulated in the dye-retention population. Other up-regulated genes were associated with EMT and secretory functions. THBS1 and other proteins enriched in LRCs can be connected to each other based on published associations and analyzed by STRING (Figure 2E). We confirmed significant up-regulation of *THBS1* by qRT-PCR performed on Dil^bright^ cells derived from four melanoma cell lines on the array. This was validated independently on two additional cell lines (LM-MEL-33 and LM-MEL-53) (Figure 2F). A gene-set enrichment analysis (GSEA) of this dataset identified gene sets associated with pertinent cell types and biological functions, notably; EMT [27-29], TGF-beta signaling [23], invasion [24, 25], and chemotherapy-resistant cancer cells [36, 37] (Supplementary Table S4).

### Thrombospondin 1 expression is associated with increased melanoma cell invasion

**Figure 3:**
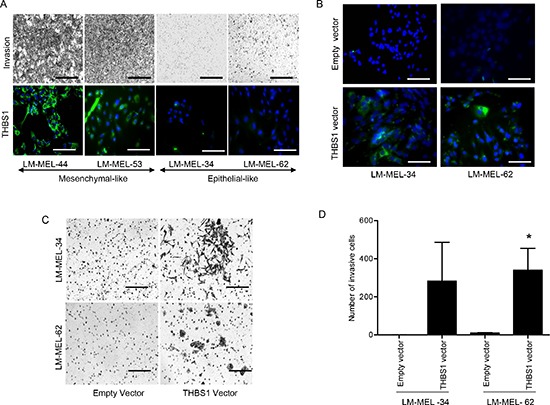
*THBS1* expression induces melanoma cell invasion (**A**) Mesenchymal- and epithelial-like melanoma cells were subjected to invasion through matrigel-coated Boyden chambers. After 48 hours cells were fixed and images of invasive cells were taken (scale bar = 500μm) or stained using THBS1 antibody in conjunction with an Alexa-488 conjugated secondary antibody and visualized with a fluorescent microscope (scale bar = 100μm). (**B**) THBS1 protein expression in two clonally derived LM-MEL-34 and LM-MEL-62 cell lines and respective empty vector control was detected using immunostaining (scale bar = 100μm). (**C**) Invasiveness of epithelial-like cells with empty vector control or *THBS-1* expression vector was determined (scale bar = 100μm). (D) The graphs show the total number of invasive cells counted. Values are mean +/− SEM of three experiments in triplicate (* p<0.05).

Invasion is an important functional change that accompanies EMT [21]. The role of *THBS1* in melanoma cell invasion was evaluated using mesenchymal- and epithelial-like melanoma cells in matrigel based transwell invasion assays. THBS1 expressing mesenchymal-like lines were highly invasive. Conversely, epithelial-like cells were less invasive and showed little or no THBS1 expression (Figure 3A).

To confirm the role of *THBS1* in invasion, a *THBS1* expression construct was generated and transfected into epithelial-like cells LM-MEL-34 and LM-MEL-62. A stable clone overexpressing *THBS1* was established for each line. Overexpression of THBS1 protein in these epithelial-like melanoma cells was confirmed by immuno-staining (Figure 3B), and greatly enhanced their invasive ability (Figure 3C&D).

### *In vitro* and *in vivo* silencing of Thrombospondin 1 inhibits invasion of melanoma cells

**Figure 4:**
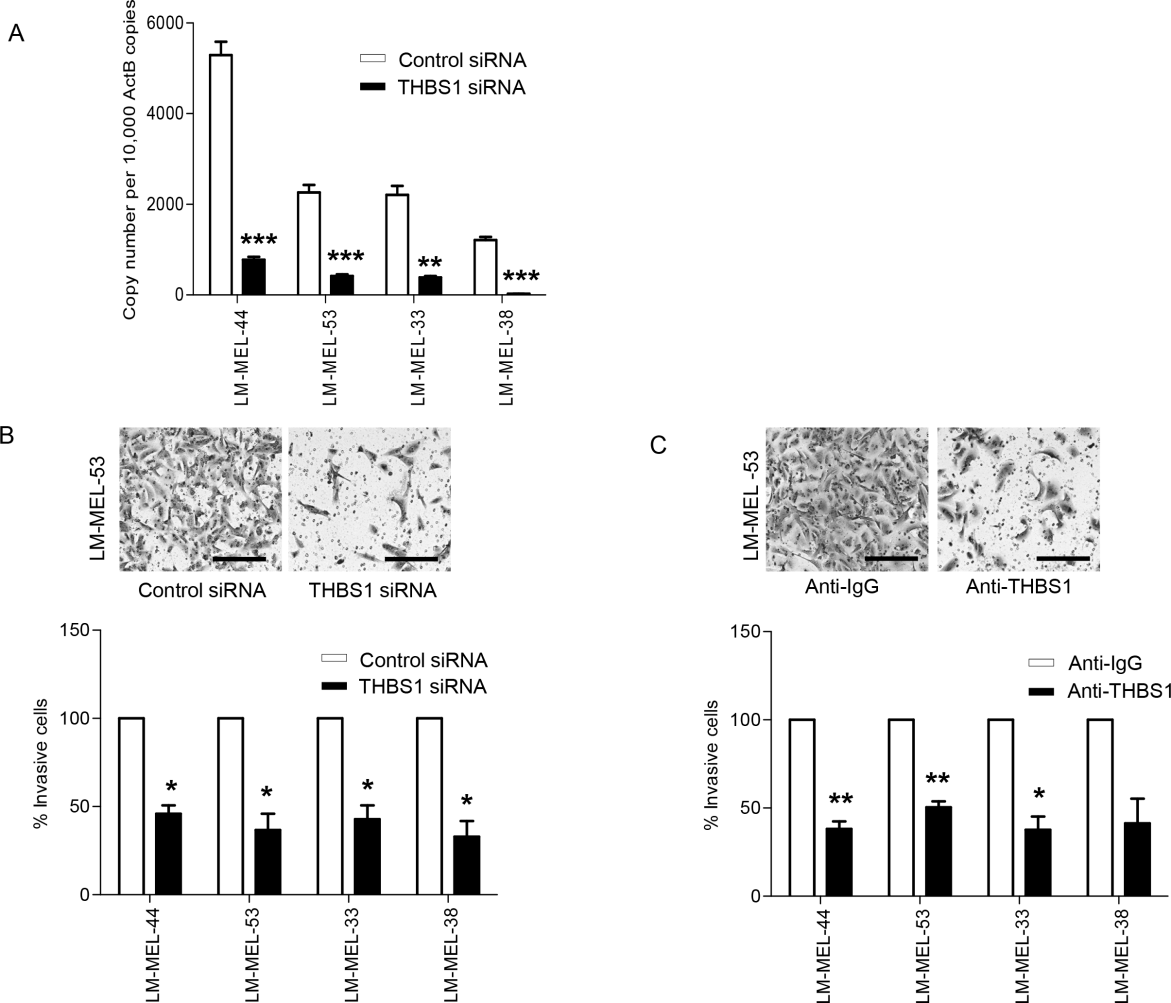
Targeting *THBS1* results in loss of invasive potential of melanoma cells *in vitro* (**A**) Melanoma cells were plated out and transfected with either 20nM control siRNA or *THBS1* specific siRNA. After 72 hours RNA was extracted and *THBS1* qRT-PCR was performed. (**B**) Melanoma cells were transfected as described and representative images of invasion in LM-MEL-53 are shown after 24 hours (scale bar = 100μm). The graphs show the total number of invasive cells counted and cell numbers from control were set to 100% and compared to *THBS1* siRNA transfected cells. (**C**) Invasiveness of melanoma cells after treatment with control anti-IgG or anti-THBS1 antibodies was tested, images captured (scale bar = 100μm) and quantified as above. Values are mean +/− SEM of four independent experiments in triplicate (* p<0.05, ** p<0.005, *** p<0.0005).

RNA interference *in vitro* with two different siRNAs was used to evaluate the role of *THBS1* in invasion. siRNA treatment silenced *THBS1* expression at mRNA level over 80% in LM-MEL−44, −53, −33 and −38 (Figure 4A). Immuno-staining confirmed reduction in THBS1 protein expression following knockdown with siRNA (Supplementary Figure S5A). ELISA also confirmed reduced secretion of THBS1 following knockdown (Supplementary Figure S5B).

Knockdown of *THBS1* in more epithelial-like melanoma cells expressing *THBS1* and some epithelial markers showed significant increase in E-cadherin levels (Supplementary Figure S5C&D). This restoration in E-cadherin levels was associated with a parallel increase in expression of *MLANA*, *TYR* and *MITF* (Supplementary Figure S5E, F&G). Analysis of the cutaneous melanoma dataset consisting of 376 melanoma patients available from TCGA revealed an increase in mRNA expression of *THBS1* in 3% of patients. This altered subset of patients showed significantly low E-cadherin protein expression compared with patients with unaltered THBS1 expression (Supplementary Figure S5H). These data confirm the inverse correlation between THBS1 expression and E-cadherin expression that was observed in our melanoma cell line studies in a large clinical tumor dataset.

Decreased invasion was observed in all cell lines transfected with *THBS1* siRNA, as compared with the control siRNA treated cells (Figure 4B). To further confirm the role of *THBS1* in melanoma invasion, melanoma cells were treated with an anti-THBS1 antibody and subjected to invasion assays. Antibody treatment decreased the number of invaded cells in all four cell lines tested (Figure 4C). Thus, loss of THBS1 contributed to the restoration of E-cadherin levels and inhibition of invasion in melanoma cells.

**Figure 5:**
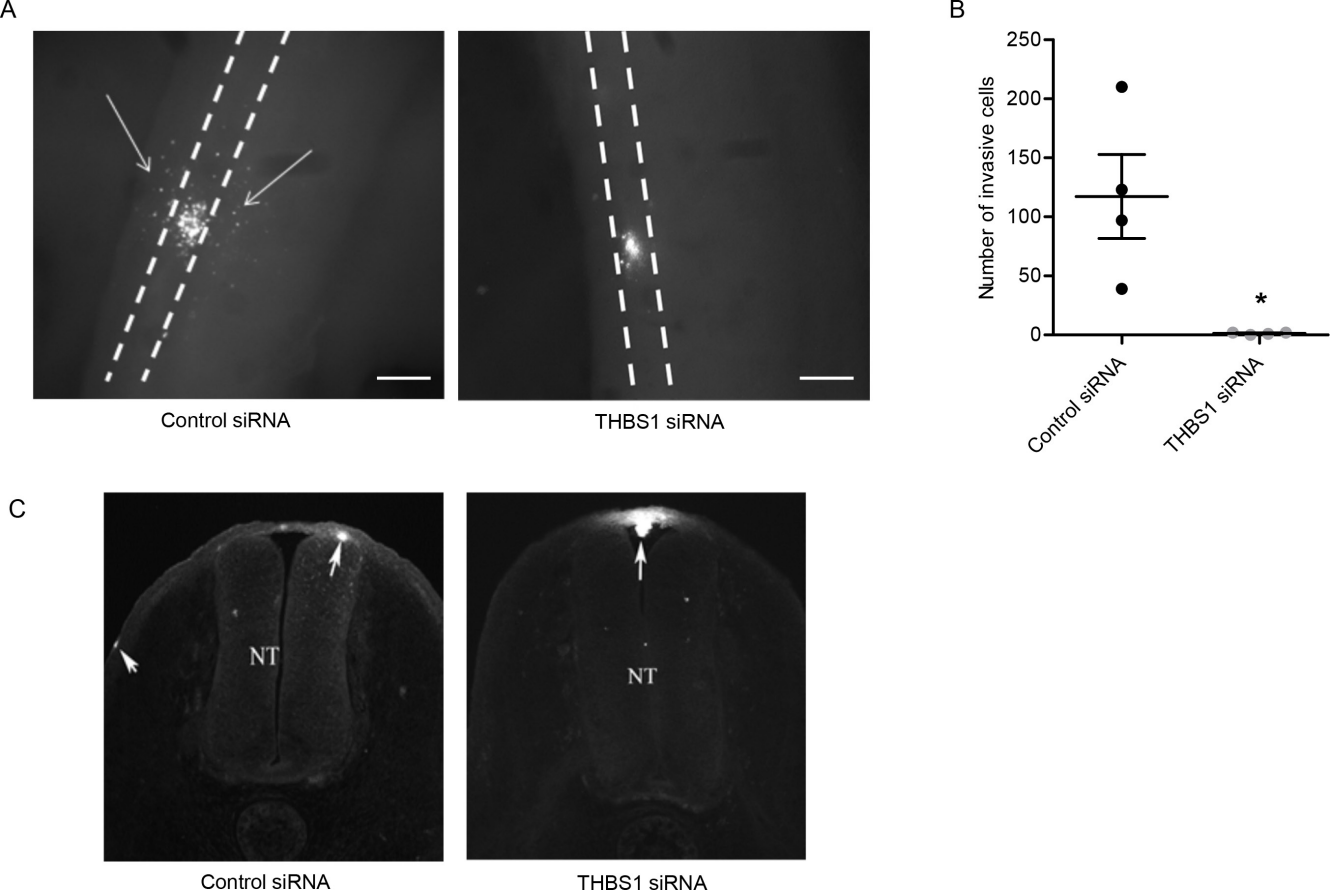
RNAi mediated loss of *THBS1* blocks motility in *in vivo* chick model Melanoma cells were treated with CM-DiO, transfected with the indicated constructs and cultured as hanging drops to encourage aggregate formation. Similar sized aggregates were introduced into the neural tube of developing chicken and re-incubated within the egg for 2 days. (**A**) Embryos were harvested and fluorescence pictures from whole-mounts taken (scale bar = 50μm). White dotted line shows the outline of the neural tube and the white arrows indicate melanoma cells that migrated out of the neural tube and into the surrounding tissue. (**B**) Analysis of cell numbers infiltrating the surrounding tissue from several independent experiments (n=4 for control siRNA and *THBS1* siRNA) (* p<0.05). (**C**) Images from cross-sections of embryos. White arrows indicate melanoma cells and NT denotes neural tube, dorsal is to the top.

We assessed *THBS1* function in an *in vivo* model of melanoma cell plasticity and invasion, the chick embryo. The model involves the injection of melanoma cells into the neural tube of developing chicken embryos where the cells acquire a motile phenotype and follow the migratory path of their ancestral cell types into the surrounding tissues. It therefore enables assessment of functions that are relevant to the metastatic process such as invasion, cellular polarity and cellular positioning [38, 39]. Melanoma cells were transfected with siRNA targeting *THBS1*, cultured as a hanging drop for 24 hours and introduced into the trunk neural tube of a developing chick embryo. *THBS1* siRNA treated cells demonstrated a significant reduction in emigration from the neural tube *in vivo* into the surrounding tissue (Figure 5A&B). Cross-sections of chick embryos confirmed that numerous control siRNA treated cells migrate out of the neural tube in contrast to *THBS1* siRNA treated cells that predominantly remain at the site of injection (Figure 5C). These results suggest a role for *THBS1* in melanoma invasion *in vivo* in response to physiological environmental signals.

### Thrombospondin 1 is upregulated in a subset of drug resistant melanoma cell lines

Drug resistance can be associated with the EMT process [4]. To examine whether *THBS1* expression was associated with the acquisition of drug resistance, six melanoma cell lines were treated with chemotherapy agents. qRT-PCR showed induction of *THBS1* expression in melanoma cells treated with cytotoxic chemotherapy agents, such as taxol, cisplatin and 5-flurouracil after 72 hours (Supplementary Figure S6A). Next, we assessed whether *THBS1* might play a role in therapies specifically targeting activated BRAF. To relate our results to findings from studies with BRAF V600E-specific inhibitors, we clustered gene expression microarray data from a publication examining mechanisms of resistance to the activated BRAF-inhibitor PLX4032 by analysing the gene set that we identified to be up-regulated in the mesenchymal-like cells (Figure 2E & Supplementary Figure S6B) [40]. Data from two of PLX4032 resistant cell lines (M229 and M238) clustered together and demonstrated up-regulation of mesenchymal-associated genes including *THBS1* in comparison to the parental cell lines (Supplementary Figure S6B). Both lines have previously been described as undergoing a change in morphology characteristic of EMT during the acquisition of resistance, whereas the cell line that did not up-regulate the LRC genes (M249) acquired resistance via a genetic mutation of NRAS and did not undergo morphological change [40]. These data show that in some cases BRAF-resistance is accompanied by an induction of *THBS1* expression and acquisition of a mesenchymal gene expression pattern.

**Figure 6:**
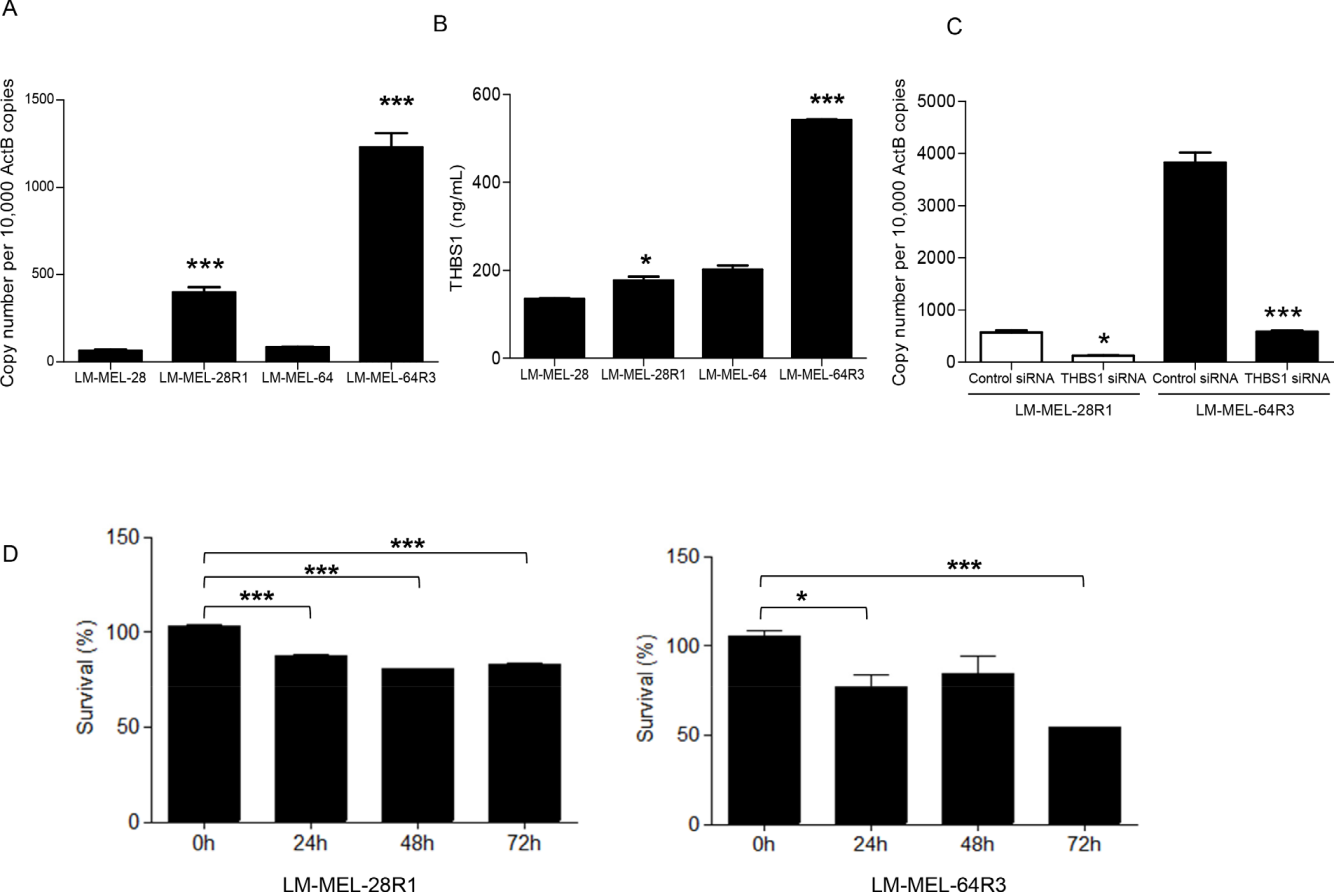
Melanoma cells surviving treatment with BRAF V600E inhibitor up-regulate *THBS1* THBS1 expression by qRT-PCR (**A**) and secretion by ELISA (**B**) in PLX4720 resistant LM-MEL-28R1 and LM-MEL-64R3 melanoma cell lines relative to their sensitive parental lines was determined. (**C**) LM-MEL-28R1 and LM-MEL-64R3 melanoma cell lines were treated with control or *THBS1* siRNA and knockdown was evaluated with qRT-PCR and (**D**) percentage survival was quantified from absorbance obtained from MTS measurement. Bars are mean values +/− SEM from three independent experiments in triplicate (* p<0.05, ** p<0.005, *** p<0.0005).

We next established two PLX4720 resistant cell lines from the V600E BRAF mutated parental lines by continuous culture in the presence of 5μM or 1μM of the drug over 10 weeks (LM-MEL-28 R1 and LM-MEL-64 R3). qRT-PCR and ELISA revealed that the expression and secretion of THBS1 was higher in LM-MEL-28R1 and LM-MEL-64R3, when compared with the drug sensitive parental cell lines (Figure 6A&B). Furthermore, depletion of *THBS1* with siRNA treatment resulted in significant restoration of drug sensitivity in the resistant cell lines (Figure 6C&D). These data show that inhibition of *THBS1* can impact on sensitivity to drugs that act via an independent signaling pathway. This raises the possibility that the THBS1/TGF-beta axis may enable drug resistance though a mechanism mediated by cellular plasticity and unrelated to the commonly described alternative MAPK signaling mechanisms.

### Thrombospondin 1 is expressed in metastatic melanoma patient tumors

**Figure 7:**
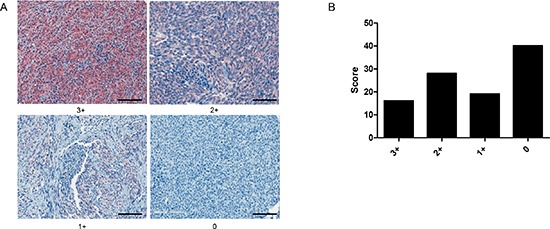
THBS1 immunostaining in melanoma tumor tissue (**A**) Localization of THBS1 in metastatic melanoma tumor biopsies. Representative tumors show score staining intensities (scale bar = 50μm). (**B**) Graph shows number of tumors scored.

THBS1 protein expression patterns in melanoma tumors were determined by immunohistochemical staining of a tissue microarrays (TMA) comprising of tumors from 103 patients with stage III and IV metastatic melanoma. THBS1 expression was detected in 61% of metastatic melanoma patient tumors (Figure 7A&B). The subcellular location was identified as predominantly cytoplasmic.

## DISCUSSION

This study examined the roles of *THBS1*, a gene which is highly expressed in mesenchymal-like but not or at lower levels in epithelial-like melanoma cell lines. *THBS1* was associated with increased invasion and drug resistance. *THBS1* expression was also increased in label-retaining melanoma cell sub-populations. Inhibition, either with neutralizing antibody or RNAi, reduced invasion of melanoma cells both *in vitro* and *in vivo*. RNAi mediated loss of *THBS1* also restored drug sensitivity in BRAF V600E mutated cells that developed drug resistance following exposure to a BRAF inhibitor.

Phenotypes similar to the epithelial- and mesenchymal-like melanoma cells described here have been demonstrated by Hoek *et al*., who classified melanoma cell lines based on invasion, high proliferation rate, and susceptibility to growth inhibition by TGF-beta treatment. Cell lines unaffected by TGF-beta were in general invasive and lost expression of MITF and associated differentiation markers. Switching between these proliferative and invasive phonotypes was proposed as a mechanism for tumor progression [41]. As these sub-populations conform to the EMT dichotomy, the Hoek phenotype switching model can be seen as a variant of EMT occurring in a non-epithelial cancer. Induction of EMT is often associated with wide-spread transcriptional changes via the activation of specific transcription factors and epigenetic regulatory mechanisms [9]. Recent research showed that a reversible EMT-TF (transcription factor) reprogramming involving upregulation of ZEB1/TWIST1 paralleled by downregulation of SNAIL2/ZEB2 occurs in human melanoma. This EMT-TF reprogramming is followed by the reduction in the expression of MITF [42] which may lead to the establishment of functional divergent melanoma cell populations based on the “rheostat model” [43]. In our system, *THBS1* is highly expressed in mesenchymal-like cells that express low *MITF* and display an invasive phenotype. This contrasts with earlier studies where low levels of *THBS1* expression have been associated with increased recurrence rates and decreased overall survival rates in several human cancers [44].

Importantly, THBS1 is a multifunctional protein with different functional and structural domains and has a variety of biological activities [15, 44]. Due to its several binding partners, the sum of downstream signals derived from the molecules occupied by the full-length protein may result in different consequences for the cell depending on the availability of THBS1 interacting proteins [45]. THBS1 has been previously described as an inhibitor of angiogenesis and tumourigenesis [17, 46] and mimetics of THBS1 have been trialled as anti-angiogenic agents [45]. It is conceivable that targeting angiogenic factors with non-redundant roles can achieve not only preventing tumor angiogenesis and metastasis, but also hindering the direct growth and invasion of tumor cells. Different approaches have been proposed to exploit protein-protein interactions in order to develop novel inhibitor peptide sequences or non-peptide, small molecule mimetics of THBS1 domains that block angiogenesis [45].

For instance, a THBS1 derived anti-angiogenic peptide ABT-510 has been tested clinically and failed to show antitumor effects in melanoma and sarcoma patients in phase II clinical trials [47, 48]. Most notably, we show a role for THBS1 in TGF-beta regulation indicating therapeutic potential beyond inhibiting angiogenesis. This may provide opportunities for therapeutic targeting despite the clinical failure of the THBS1-derived anti-angiogenic peptide. As with a wide range of molecules in cancer, an anti-angiogenic molecule could well lead to hypoxia in tumors and as a consequence the induction of an aggressive phenotype resulting ultimately in de-differentiation and metastasis [49-52].

*THBS1* can be epigenetically silenced in melanomas, marking it as a potential tumor-suppressor gene [46]. Contrasting, *THBS1* has been described as an immune modulator, both through the activation of regulatory T cells, and by facilitating the activation of TGF-beta, an immunosuppressive cytokine. A recent study showed that the transcription factor *SNAIL* induces EMT in melanoma and leads to an increase in *THBS1* expression and results in immunosuppression and enhanced metastasis [16]. Similarly, we demonstrated that *in vivo THBS1* knockdown abrogates the invasive/plastic potential of melanoma cells within the chicken neural tube. The avian embryo has emerged as a useful site for analysing melanoma cell behaviour within a developmental microenvironment since it provides imaging and surgical accessibility to manipulate and monitor the transplanted tumor [53-55]. Additionally, the biology of cells within the neural crest is well understood and since melanocytes are neural-crest-derived cells, it is likely that the environmental signals in this model are pertinent to melanoma cell motility. Thus the interaction between melanoma and motile embryonic neural crest cells provide a functional context, and thereby a valuable model for interrogating the molecular regulation of migrating melanoma [38, 39].

Although we have clearly demonstrated that *THBS1* mediates enhanced melanoma invasion *in vitro* and *in vivo*, previous studies that evaluated the role of *THBS1* in cancer cell invasion have yielded mixed results. Although *THBS1* expression was inversely correlated with cell invasion in thyroid cancer in one study [56], other studies reported that *THBS1* promotes cell invasion in breast cancer, thyroid cancer, colon cancer and prostate tumors [57-59]. In addition, knock-out of *THBS1* in an animal model of breast cancer led to growth of the primary tumor but a decrease in the number of metastases [61]. Thus, the pathological roles and clinical significance of *THBS1* may depend on the nature of the model and on the stage of tumor progression. To better evaluate role of *THBS1* in human melanoma, we determined its expression in metastatic melanoma tumor biopsies. THBS1 protein was located in tumor cells and was undetectable in stroma. This contrasts with gastric cancer, where THBS1 localized to stroma [62], indicating apparent tissue-specific differences depending on cancer type.

The mechanism whereby *THBS1* promotes melanoma invasion remains poorly understood, however we demonstrate that this is likely linked to TGF-beta activation. TGF-beta 1 is secreted in a biologically inactive latent form and activation of this latent TGF-beta 1 is required for biological activity [19]. *THBS1* has been identified as a major physiological activator of TGF-beta 1 both *in vivo* and *in vitro* [18, 63]. The interaction between tripolymer THBS1 and latent TGF-beta 1 causes a conformational change in the latent complex and exposes epitopes that are critical for binding to the cellular receptor. Our data show that THBS1 and TGF-beta 1 is expressed and secreted by mesenchymal-like melanoma cells and further, that *THBS1* is inducible in epithelial-like cells upon exposure to TGF-beta 1. The interplay between these molecules supports a role for positive feedback within the tumor and extends the observations of others that TGF-beta 1 induces EMT in melanoma [21, 31] and that *THBS1* is a TGF-beta 1 activator [18]. We propose that *THBS1* promotes EMT in melanoma through activation of latent TGF-beta 1 during the progression of melanoma.

Finally we demonstrate a striking association between the expression of *THBS1* and drug resistance, not only to cytotoxic drugs, but also in melanoma cells resistant to BRAF inhibition. The list of genetic and non-genetic changes that are associated with resistance to the targeted MAPK inhibitors continues to grow and our data adds to this with the clear demonstration that *THBS1* can play a functional role in BRAF inhibitor resistance. Although a direct mechanism cannot readily be implied from an understanding of its various biological specificities, an indirect role can readily be explained via TGF-beta-induced EMT, or through association with the LRC phenotype. Slow cycling cells have recently been associated with BRAF inhibitor-resistance and melanoma invasiveness [13]. In our studies *THBS1* expression in LRC was associated with an EMT-like phenotype and potentially implicates TGF-beta pathway activation in LRC to mediate tumor progression. These findings contrast with those of Roesch *et al*., who did not identify EMT possibly explained by differences in the experimental systems utilized. Nonetheless it is clear that further studies of LRC, *THBS1* and the role of TGF-beta signaling will be critical to better understand heterogeneity, plasticity and the mediators of phenotype switching as contributors to treatment failure. As a central player in these processes, *THBS1* well may serve as a target in strategies to better treat malignant melanoma.

## MATERIAL AND METHODS

### Cell culture

Melanoma cell lines were established from resected melanoma metastases by mechanical dissociation of tissue with subsequent overnight digestion in media containing collagenase IV at 37°C. Established cell lines were Mycoplasma-tested using the MycoAlert test (Lonza Rockland, Inc., USA). All tissue donors provided written informed consent for tissue collection and research, which was covered by protocols approved by the Austin Health Human Research Ethics Committee, Melbourne, Australia (approval number H2012/04446). All cell lines were matched with their donors by HLA-typing. Cells were cultured in RPMI1640 supplemented with 10% fetal calf serum (FCS) as described previously [22].

### qRT-PCR

RNA for qPCR was extracted using the RNEasy kit (Qiagen, Germany) or the Acturus^®^ RNA Picopure^®^ kit (Life Technologies, USA). Reverse transcription was carried out using the High Capacity cDNA RT kit (Applied Biosystems, Life Technologies, USA). Following reverse transcription, qRT-PCR was performed using SYBR Green (Qiagen, Germany). Beta-Actin (*ActB*) was used as internal control. Following primers were used: *ActB* (forward) 5’-ctg gaa cgg tga agg tga ca-3’ and (reverse) 5’-cgg cca cat tgt gaa ctt tg- 3’, E-cadherin (forward) 5’-gcc gag agc tac acg ttc a-3’ and (reverse) 5’-gac cgg tgc aat ctt caa a-3’, N-cadherin (forward) 5’-ctc cat gtg ccg gat agc-3’ and (reverse) 5’-cga ttt cac cag aag cct cta c -3’, *THBS1* (forward) 5’- caa tgc cac agt tcc tga tg-3’ and (reverse) 5’-tgg aga cca gcc atc gtc-3’, *TYR* (forward) 5’-gct gcc aat ttc agc ttt aga -3’ and (reverse) 5’-ccg cta tcc cag taa gtg ga -3’, *MLANA* (forward) 5’-gag aaa aac tgt gaa cct gtg gt -3’ and (reverse) 5’-gac tgt tct gca gag agt ttc tca t -3’, *MITF* (forward) 5’-cat tgt tat gct gga aat gct aga -3’ and (reverse) 5’-tgc taa agt ggt aga aag gta ctg c -3’.

### Gene-expression arrays and data analysis

RNA was analysed on Illumina HT-12 v3 arrays at the Australia Genome Research Facility (AGRF, Australia). Raw data were read in to the R environment for statistical computing (http://www.r-project.org/) using the limma package [64], background was corrected using the normexp function, and log2 transformed and quantile normalized. Differential expression for the cell line panel was determined using the ANOVA tool in Partek Genomics Suite (Partek GS). Differential expression for the smaller label-retaining data set employed the RankProd package for differential expression analysis, using a percentage of false positive (PFP – approximation of false discovery rate (FDR)) cut-off of 5% to determine statistical significance [65]. Gene-set enrichment analysis (GSEA) settings were; gene set permutation, a FDR of 5%, and the MSigDB 4.0 database (category C2). Lists of differentially expressed genes were used to create network diagrams in STRING [66], with connections between molecules based on different levels of molecular interaction evidence, such as known physical interaction, correlated gene expression, and literature mining. Microarray data from Nazarian R *et al.* were obtained from NCBI GEO (accession GSE24862). Affymetrix CEL files were normalized by RMA [67] in Partek GS. Principal components analysis and hierarchical clustering of all datasets were performed in Partek GS, with hierarchical clustering using average linkage and Pearson's dissimilarity as a distance metric.

### Solid phase ELISAs

THBS1 and TGF-beta 1 protein amount in cell line supernatants was measured by Quantikine human Thrombospondin 1 (R&D Systems, USA) or TGF-beta1 immunoassay kit (Life Technologies, USA) as per the manufacturer's instructions.

### TGF-beta 1 treatment

LM-MEL-42 and LM-MEL-34 was treated with 5ng/ml TGF-beta 1 (Pepro Tech Inc.,USA). RNA was extracted 24, 48 and 72 hours following treatment.

### VybrantDye labeling

LM-MEL-44, LM-MEL-28, LM-MEL-33, LM-MEL-34, LM-MEL-42 and LM-MEL53 were stained with 5μl of VybrantDye CM-Dil (Life technologies, USA) for 25min at 37°C/5% CO_2_.

### Flow cytometry and sorting

Melanoma cell lines were stained with Pacific-Blue (Life Technologies, USA) according to the manufacturer's protocol and flow cytometry was performed using a FACSCanto II. Cell sorting utilized a FACSAria III (all Becton, Dickinson and Company, USA).

### Animal experiments

Ethical approval was provided by the Austin Health Animal Ethics Committee. Ascites cells from the peritoneal fluid of a patient with advanced stage IV melanoma were confirmed as melanoma by a pathologist in the Austin Hospital. Red blood cells and debris were removed with Ficoll-Plaque centrifugation. Cells were stained with CM-Dil, resuspended in PBS and injected subcutaneously in mice (n=3). Tumor growth was monitored three times a week, and mice were sacrificed after three weeks. Tumors were removed and paraffin-embedded.

### Invasion assay

Invasion assays were performed in Boyden chamber inserts with Matrigel coating (Becton, Dickinson and Company, USA). Insert membranes were stained with 4', 6-diamidino-2-phenylindole (DAPI) or a 0.1% crystal violet solution (Sigma, USA). Cells were photographed with a monochromatic Olympus camera. The total number of invaded cells was counted from three representative fields of view per chamber, at 10× magnification.

### *THBS1* over-expression construct

The plasmid encoding *THBS1* was obtained from Sino Biological Inc. Cells were transfected using Lipofectamine 2000 (Life Technologies, USA). Stably transfected cells were selected for and maintained in media containing 0.6mg/ml G418, with colonies isolated using cloning cylinders (Merck, USA).

### *THBS1* knockdown and immunofluorescence

For transient siRNA transfection, cells at 30% confluence were transfected using a control siRNA and two different Silencer select siRNAs targeting *THBS1* (s14100 and s14098) at 20nM final concentration (Ambion, USA) with Lipofectamine RNAiMAX according to the manufacturer's protocol (Invitrogen, USA). Cells were incubated with siRNA complex for 48 hours and then fixed with 4% paraformaldehyde, stained with anti-THBS1 antibody (A6.1, NB100-2059, Novus Biologicals, USA) was applied at 2.5μg/mL concentration overnight at 4°C and with Alexa flour 488 conjugated secondary antibody for 45 minutes at room temperature (Molecular probes, USA). Cells were counter stained with DAPI for 10 minutes.

### *In vivo* chick embryo model

Melanoma cells were treated with *THBS1*-specific siRNAs or scrambled control siRNA as described and labelled with CM-DiO or Dil as per manufacturer's instructions (Invitrogen, USA). Cells were grown overnight in a hanging-drop fashion to allow the formation of aggregates. Fertile chicken eggs were incubated at 38°C for 48 hours prior to transplantation. Cell aggregates consisting of 50-400 cells were harvested and carefully injected with a glass pipette into the trunk neural tube lumen of developing chicken embryos. The eggs were then sealed with adhesive tape and re-incubated for 2 days. After incubation, embryos were removed from the eggs and fixed with 4% paraformaldehyde and whole mounts or cross sections were analyzed for the localization of melanoma cells using Lumar V12 Zeiss microscope.

### Treatment with chemotherapy agents

Melanoma cells were treated with cytotoxic chemotherapy agents, such as Taxol (40nM), 5-Flourouracil (150μM) or Cisplatin (1μM) or DMSO (all Sigma Aldrich Pty Ltd., USA) for 72 hours.

### Generation of PLX4720 resistant cell lines

LM-MEL-28 and LM-MEL-64 were grown in media with 5μM or 1μM PLX4720 (Scientifix Pty Ltd, Australia) for 10 weeks; media was exchanged every third day. Parental cell lines treated with DMSO served as control. Cells for RNA extraction were harvested at 80% confluency.

### Proliferation assays

10,000 cells per well were plated out in 96 well plates and treated as indicated. Relative cell numbers were measured using the CellTiter 96^®^ AQueous One Solution Cell Proliferation Assay (Promega Corporation, USA).

### Immunohistochemistry and pathological evaluation

Paraffin embedded tissue slides were deparaffinised and rehydrated, endogenous peroxidise activity was blocked with 3% Hydrogen peroxide, antigen retrieval was performed in 10mmol/L citrate buffer, and nonspecific binding was blocked with blocking reagent. THBS1 antibody (A6.1, NB100-2059, Novus Biologicals, USA) was applied at 6μg/mL concentration and incubated overnight at 4°C, followed by 60 minute incubation with secondary anti-mouse antibody HRP (Dako). The chromogen used was 3-amino-9-ethylcarbazole (AEC). Human placenta was used as the positive control for THBS1 and a negative control, for which the primary antibody was substituted with the same concentration of mouse IgG. Slides were scanned using a ScanScope XT (Aperio) and immunohistochemical reactivity was evaluated by two independent investigators. The expression of THBS1 was categorized into four grades. They were arbitrarily scored as 3, strong staining; 2, moderate staining; 1, weak staining and 0, no staining.

### Statistical analysis

All statistical comparisons of data sets were performed using Student's two-tailed t-test in Prism software version 5.00 (GraphPad Software Inc).

## SUPPLEMENTARY FIGURES AND TABLES


