# Supplementary material for: Thrombospondin 1 promotes an aggressive phenotype through epithelial-to-mesenchymal transition in human melanoma
Source: Oncotarget. 2014 Jul 8;5(14):5782–97. doi: 10.18632/oncotarget.2164 (PMC4170613; doi:10.18632/oncotarget.2164)
Supplement: Supplementary file 1 [file oncotarget-05-5782-s001.pdf]

SUPPLEMENTARY FIGURES AND TABLES

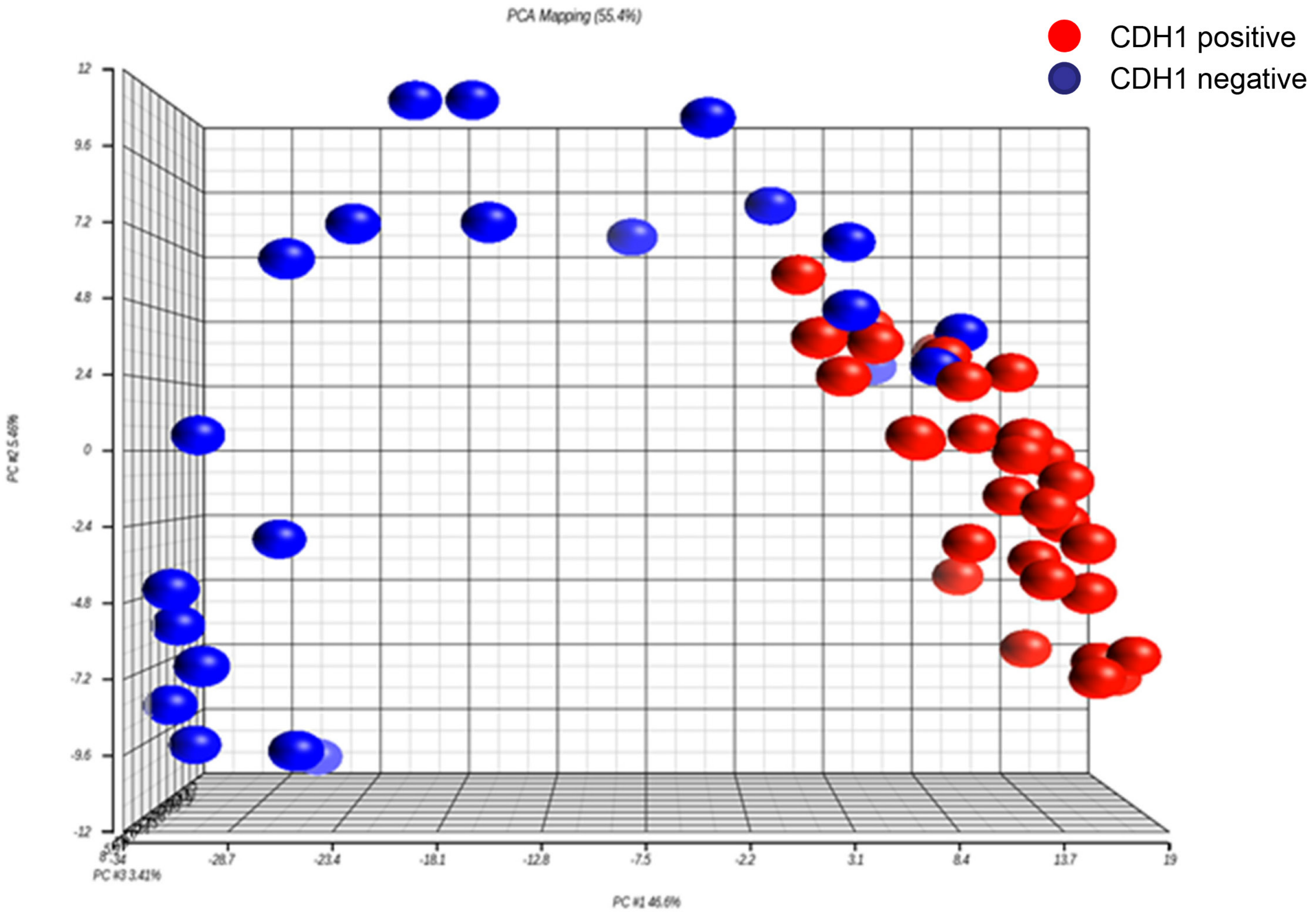

Supplementary Figure S1: Segregation of melanoma cell lines based on differential gene expression. Principal components analysis of the genes differentially expressed between 54 melanoma cell lines divided based on presence or absence of E-cadherin (*CDH1*) expression by qRT-PCR.

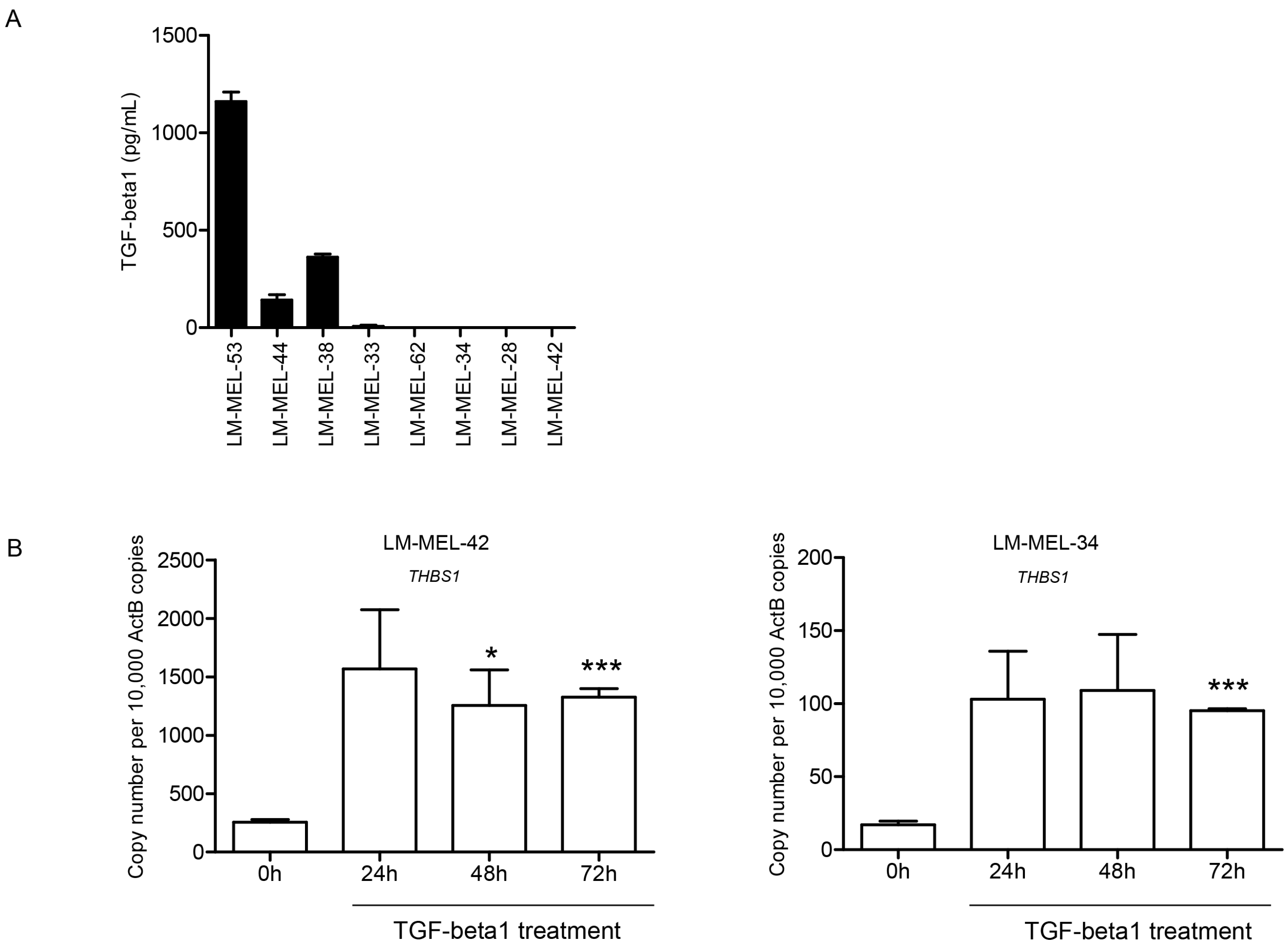

Supplementary Figure S2: TGF-beta 1 secretion and expression in melanoma cells. (A) Media from 4 mesenchymal- and 4 epithelial-like melanoma cells were collected and subjected to TGF-beta 1 ELISA, and only mesenchymal-like lines secreted TGF-beta 1. **(B)** *THBS1* expression was assessed by qRT-PCR in 2 epithelial-like melanoma cell lines; LM-MEL-42 and -34 treated with 5ng/ml of TGF-beta 1 for 24, 48 or 72 hours. Bars are mean values +/– SEM from experiments in triplicate (* $p<0.05$, *** $p<0.0005$).

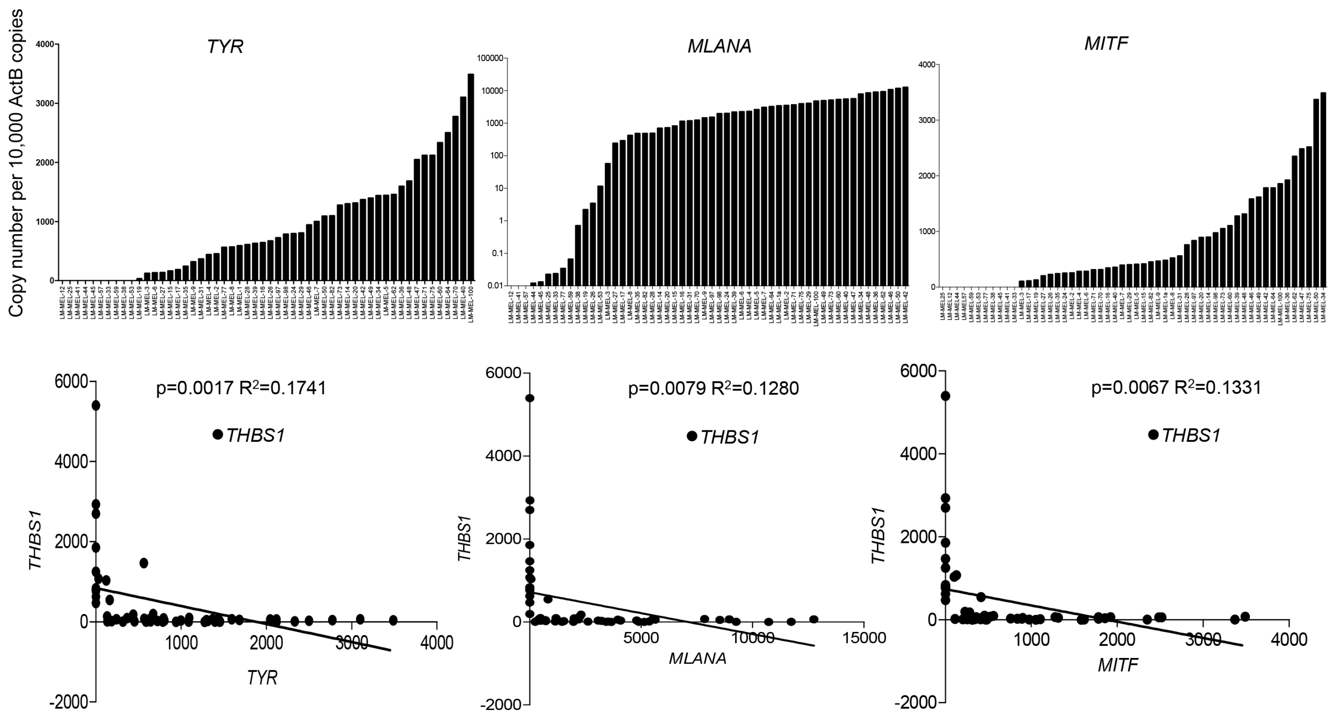

Supplementary Figure S3: Correlation between *THBS1* and differentiation marker mRNA expression in melanoma cells. qRT-PCR analysis of *TYR*, *MLANA* and *MITF* in 54 melanoma cell lines. Regression analysis of all the three genes with *THBS1* expression is shown.

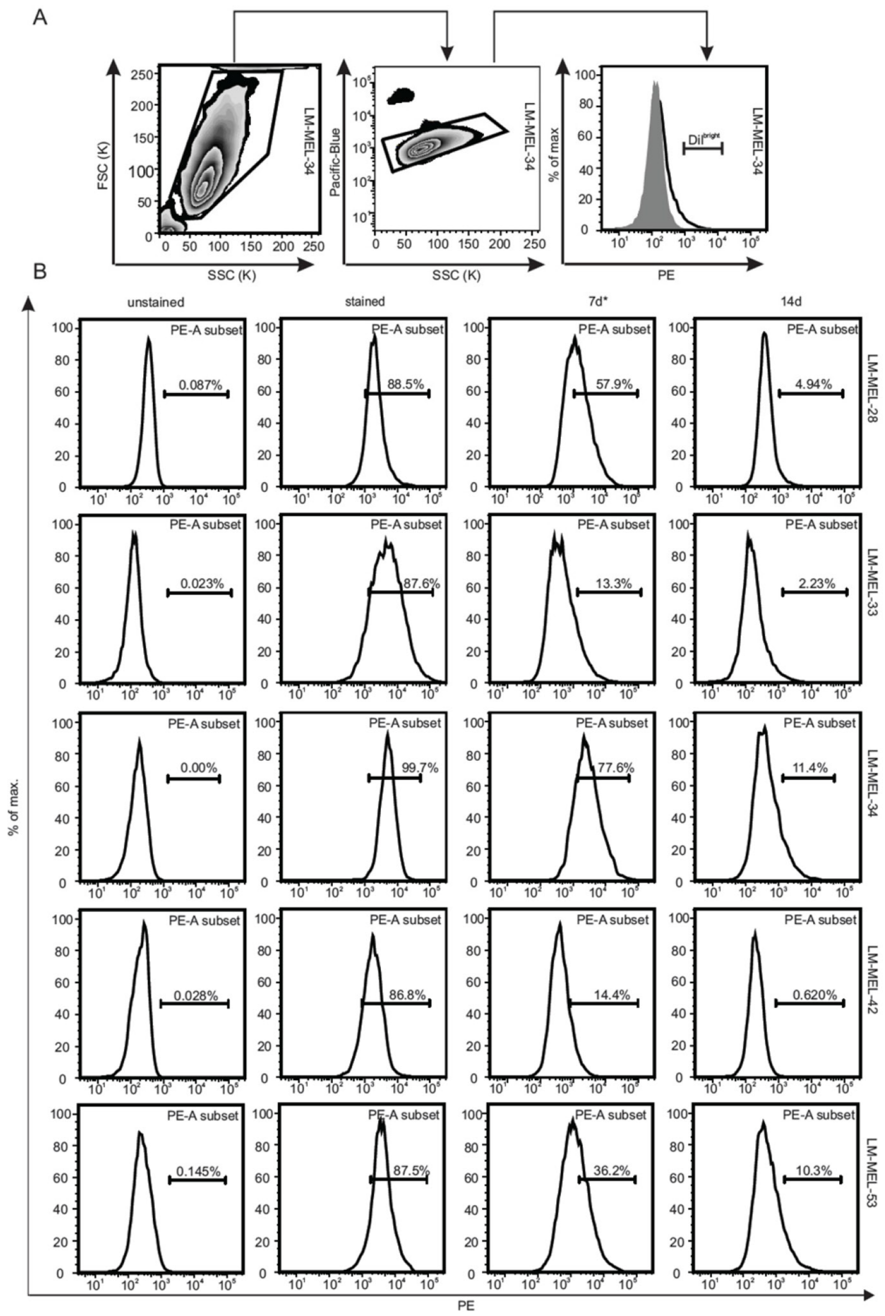

Supplementary Figure S4: Flow profile of the LRC population in different melanoma cell lines. **(A)** Gating strategy for detection of the Dil^{bright} population. Cell lines were CM-Dil labeled, cultured and gated based on FSC/SSC; dead cells were excluded using Pacific-Blue viable cell stain and then gated compared to unstained cells (from left to right). **(B)** The indicated melanoma cell lines were stained with CM-Dil and cultured for the indicated time-frame. FACS analysis was performed either with unstained cells (unstained), two hours after labeling (stained), after seven days (7d) or after fourteen days (14d). The * indicates that LM-MEL-33 histograms have been derived after 8 days. All cell lines were co-stained with Pacific-Blue to exclude dead cells from the analysis.

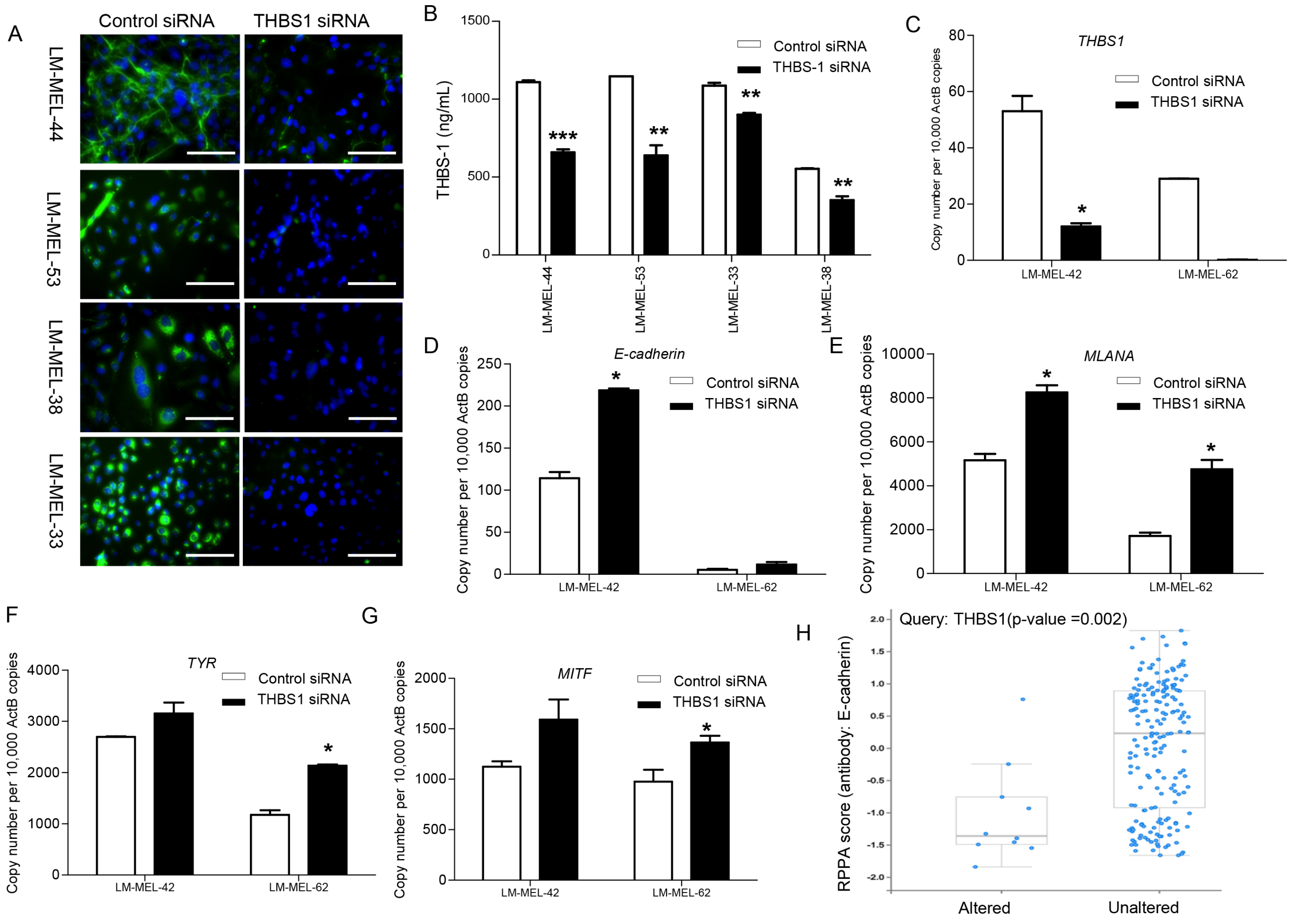

Supplementary Figure S5: Loss of *THBS1* effects E-cadherin and differentiation marker expression. Melanoma cells were plated out and transfected with either 20nM scrambled siRNA or THBS1 specific siRNA. **(A)** After 48 hours cells were fixed and stained using a THBS1 antibody in conjunction with an Alexa-488 conjugated secondary antibody (scale bar = 100µm). **(B)** Simultaneously treated cells were used for THBS1 ELISA. Epithelial-like melanoma cell lines LM-MEL-42 and LM-MEL-62 were treated with *THBS1* siRNA and mRNA expression level of *THBS1* **(C)**, E-cadherin **(D)**, *MLANA* **(E)**, *TYR* **(F)** and *MITF* **(G)** were assessed. Bars are mean values +/– SEM from three independent experiments in triplicate (* p<0.05, *** p<0.0005). (H) TCGA obtained dataset demonstrating E-cadherin protein levels in THBS1 altered (high) and unaltered (lower) melanoma patient samples.

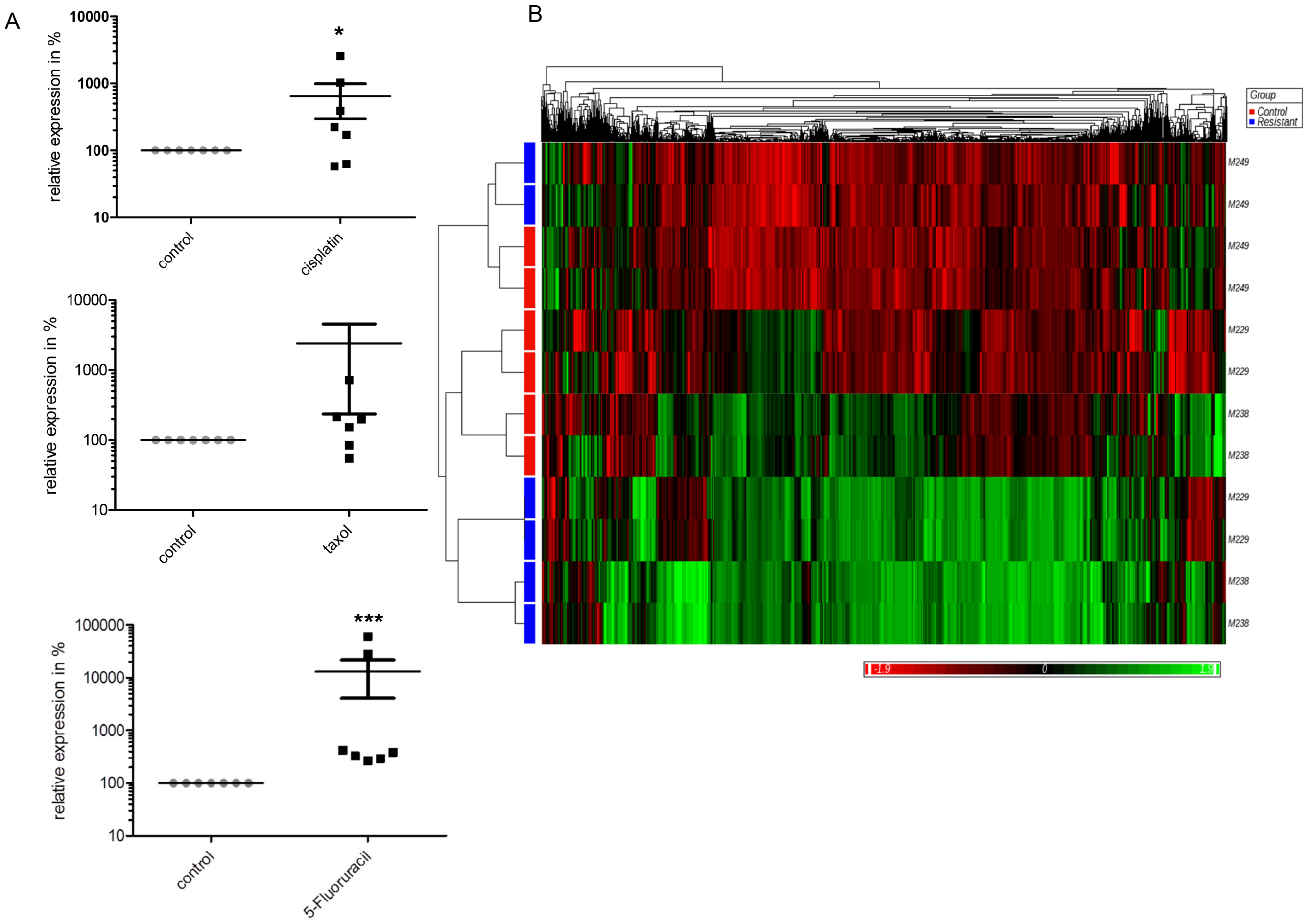

Supplementary Figure S6: *THBS1* is enriched in melanoma cells with acquired anti-tumor drug resistance and BRAF inhibitor resistance. (A) RNA was extracted from melanoma cell that survived after a three-day treatment with 40nM taxol, 150µM 5FU or 1µM cisplatin or DMSO (control) and *THBS1* expression was performed by qRT-PCR. Bars show percentage of expression +/− SEM compared to control (* $p<0.05$, *** $p<0.0005$). **(B)** Comparison of a publically available dataset (referenced in the text) of PLX4032 resistant BRAF V600E melanoma cell lines and their sensitive parental lines, based on the gene up-regulated in mesenchymal-like melanoma cells (Supplementary Table S1). *THBS1* was significantly up-regulated in the M229 (mean 2.34 fold) and M238 (mean 1.68 fold) resistant lines compared to the parental lines (*THBS1* probe IDs 7982599 and 7982600; ANOVA FDR cut-off < 5%). M249 did not show a change in expression for *THBS1*.

Supplementary Table S1.

Symbol	Probe ID	p-value	Fold-Change
LOC100134134	ILMN_337630	1.74E-07	32.11
AXL	ILMN_28087	1.23E-07	22.47
THBS1	ILMN_182705	5.53E-07	18.90
AXL	ILMN_180683	8.51E-08	18.11
PXDN	ILMN_165066	3.00E-07	16.40
FAM20C	ILMN_5460	1.09E-07	13.33
COL5A1	ILMN_31902	5.34E-07	13.24
CCL2	ILMN_25185	9.67E-07	13.15
NTM	ILMN_169728	6.17E-10	11.06
C20orf100	ILMN_17741	3.25E-05	10.70
TPM2	ILMN_22618	0.000129141	10.18
NRP1	ILMN_17483	1.20E-07	9.98
HLA-B	ILMN_18149	2.68E-06	9.87
ID1	ILMN_28002	4.71E-07	9.72
IGFBP6	ILMN_5216	9.40E-10	9.66
EFEMP1	ILMN_41104	8.65E-08	9.55
C5orf13	ILMN_2442	1.76E-08	9.51
COL6A1	ILMN_179738	2.35E-07	9.44
BASP1	ILMN_28962	1.12E-05	9.34
DKK3	ILMN_4127	0.000355612	9.19
FOXD1	ILMN_182299	2.57E-09	9.15
COL6A2	ILMN_21525	7.00E-08	9.09
COL8A1	ILMN_10408	1.91E-05	8.75
DKK3	ILMN_181104	0.000309747	8.44
TPM2	ILMN_22618	0.00016883	8.34
PMEPA1	ILMN_13834	8.93E-07	8.29
LOC652683	ILMN_36509	7.05E-05	8.17
SPANXA2	ILMN_28893	0.000124942	7.91
SPANXB1	ILMN_23554	0.000118767	7.83
SFRP1	ILMN_21487	0.000280032	7.81
PMEPA1	ILMN_13834	6.95E-06	7.81
CDH2	ILMN_28694	6.21E-06	7.42
JUN	ILMN_7746	7.70E-09	7.39
TM4SF1	ILMN_6834	0.000306286	7.08
WNT5A	ILMN_164155	1.11E-06	6.94
HS3ST3A1	ILMN_21069	2.41E-05	6.75
NDRG1	ILMN_24220	2.65E-05	6.65

Symbol	Probe ID	p-value	Fold-Change
VCAN	ILMN_25778	3.07E-05	6.64
NPTX1	ILMN_5048	0.000220168	6.57
IFITM3	ILMN_6890	7.19E-05	6.46
LOC100133171	ILMN_346604	0.000103143	6.37
PODXL	ILMN_24120	9.93E-05	6.36
LOC387882	ILMN_23241	2.78E-06	6.32
IRF1	ILMN_11739	3.38E-06	6.24
BGN	ILMN_6146	0.000142392	6.22
FER1L3	ILMN_18562	2.98E-07	6.16
PLAU	ILMN_24167	0.000114489	6.15
FAM171A1	ILMN_17888	5.01E-06	6.03
TPM1	ILMN_14091	9.29E-06	5.95
LEPREL1	ILMN_11123	0.000183491	5.89
CAV1	ILMN_178269	1.39E-06	5.89
NPTX2	ILMN_22638	0.000120175	5.89
SPOCK1	ILMN_25886	0.000204002	5.85
SERPINE1	ILMN_6244	1.90E-05	5.82
IL1RAPL1	ILMN_7989	0.000444637	5.79
COL6A2	ILMN_21525	3.39E-07	5.73
TPM1	ILMN_8136	1.07E-05	5.71
ADAM19	ILMN_12727	0.000461085	5.69
SPANXA1	ILMN_9452	0.000125831	5.67
MYOF	ILMN_371188	3.76E-06	5.57
LPAR1	ILMN_28278	4.03E-06	5.57
RUNX1	ILMN_19125	1.13E-07	5.57
SPANXC	ILMN_1520	0.00013928	5.45
PRKCA	ILMN_24085	3.21E-07	5.40
KRT80	ILMN_3629	1.34E-05	5.39
FER1L3	ILMN_28913	6.59E-06	5.34
NRP1	ILMN_16360	8.43E-07	5.34
GLIPR1	ILMN_3517	2.53E-05	5.32
GBP2	ILMN_183366	0.000134175	5.29
GAD1	ILMN_4673	1.67E-05	5.26
KIAA1644	ILMN_45890	1.12E-05	5.24
ERRFI1	ILMN_4328	0.00021487	5.22
TOX2	ILMN_308609	1.85E-05	5.22
VASN	ILMN_31069	3.50E-05	5.21
RIN2	ILMN_17838	5.63E-07	5.20

(*Continued*)

Symbol	Probe ID	p-value	Fold-Change
IFITM2	ILMN_2038	0.000193514	5.18
TLE4	ILMN_14046	1.73E-07	5.16
FZD2	ILMN_12499	4.56E-07	5.12
TAGLN	ILMN_2335	0.000661612	5.05
IL1B	ILMN_27277	0.000164867	5.02
LOC399959	ILMN_371215	4.16E-06	4.98
EFNB2	ILMN_3827	2.89E-06	4.96
IL6	ILMN_6469	0.000405885	4.91
VEGFC	ILMN_161973	1.10E-05	4.90
PTRF	ILMN_22301	0.000488678	4.80
NNMT	ILMN_22529	0.000937969	4.79
KCNMA1	ILMN_24236	2.43E-06	4.76
TFPI	ILMN_17834	1.77E-05	4.75
SPANXB2	ILMN_2518	0.00091498	4.73
ADAMTS1	ILMN_11081	1.47E-05	4.71
GAS6	ILMN_10723	0.00050824	4.68
COL8A1	ILMN_10408	7.62E-05	4.68
AFAP1	ILMN_5058	4.35E-06	4.68
TMEM158	ILMN_13668	0.000335444	4.64
ITGA5	ILMN_165321	0.000776225	4.64
EFEMP2	ILMN_42286	7.02E-05	4.64
COL7A1	ILMN_24830	0.000496306	4.64
MYLK	ILMN_11781	9.18E-06	4.63
APCDD1L	ILMN_29515	0.000251296	4.54
FLJ40504	ILMN_176567	2.40E-05	4.45
GAS6	ILMN_10723	0.000627717	4.45
TFPI	ILMN_17834	2.22E-05	4.45
ANTXR2	ILMN_165233	0.000158199	4.39
NUAK1	ILMN_12806	0.000231994	4.39
IL7R	ILMN_137414	9.87E-06	4.38
SPANXE	ILMN_3888	0.000170825	4.34
LDOC1	ILMN_8953	6.82E-05	4.33
KRT18P13	ILMN_344563	9.74E-06	4.30
CDR2L	ILMN_26231	0.000133274	4.30
CPA4	ILMN_164642	0.00036069	4.29
PRSS23	ILMN_22272	4.79E-05	4.29
PITPNC1	ILMN_12660	2.81E-05	4.28
PTPRE	ILMN_23422	1.46E-05	4.21

Symbol	Probe ID	p-value	Fold-Change
SLFN11	ILMN_2517	0.000389881	4.15
BCL3	ILMN_17874	7.06E-07	4.14
TNFRSF21	ILMN_9651	9.09E-05	4.11
	ILMN_92725	0.000436828	4.11
STEAP3	ILMN_28415	8.03E-06	4.10
COLEC12	ILMN_4310	0.00040579	4.09
NTM	ILMN_169728	2.43E-07	4.06
SPANXE	ILMN_3888	0.000190895	4.03
ANXA1	ILMN_14184	0.000135662	4.00
CARD10	ILMN_28765	4.64E-05	3.98
CRIM1	ILMN_10588	9.45E-06	3.90
GBP1	ILMN_28413	7.99E-05	3.89
SMAD3	ILMN_16351	1.13E-05	3.89
IRAK2	ILMN_174255	4.21E-06	3.88
TSPAN5	ILMN_8032	4.22E-05	3.87
MXRA5	ILMN_29922	0.000136814	3.84
KCNG1	ILMN_28652	0.000313746	3.77
SCG5	ILMN_6950	0.000971664	3.75
IFIT2	ILMN_28123	0.000107131	3.74
ARHGAP23	ILMN_162296	4.43E-05	3.74
TGFB1I1	ILMN_27048	0.00028205	3.72
SLC2A4RG	ILMN_11640	3.49E-05	3.71
TMEM47	ILMN_2011	2.92E-06	3.71
SPOCD1	ILMN_6339	0.000205229	3.68
PVRL3	ILMN_2284	0.000197657	3.66
S100A3	ILMN_19000	1.13E-06	3.65
C1orf133	ILMN_370905	0.000215443	3.62
ITGA11	ILMN_26949	3.58E-05	3.61
LARP6	ILMN_25584	7.18E-05	3.61
FLNC	ILMN_167330	0.000539122	3.59
ITGA2	ILMN_12662	1.26E-05	3.58
PDGFC	ILMN_13763	0.000113346	3.57
PAPPA	ILMN_177957	0.000383103	3.51
NUAK1	ILMN_12806	2.43E-05	3.48
HEG1	ILMN_306841	0.000204586	3.48
GPX8	ILMN_364670	2.18E-06	3.46
SDC4	ILMN_24480	6.54E-05	3.45

(*Continued*)

Symbol	Probe ID	p-value	Fold-Change
RARRES3	ILMN_1164	0.000219272	3.45
SORBS3	ILMN_23885	0.000224004	3.44
NCAM1	ILMN_7059	0.000251405	3.43
SMAGP	ILMN_22631	0.000678242	3.41
RRAS	ILMN_23748	5.37E-06	3.41
PVRL3	ILMN_2284	0.000291111	3.37
TNFRSF11B	ILMN_6495	0.000356991	3.35
DSE	ILMN_14589	9.43E-05	3.34
	ILMN_71954	0.000225806	3.34
TMEM45A	ILMN_30168	6.03E-05	3.32
SOX9	ILMN_17599	0.000682599	3.30
SLC10A3	ILMN_162950	4.17E-07	3.29
SRPX2	ILMN_23854	5.48E-05	3.29
DUSP1	ILMN_20700	0.000711119	3.29
PSMB9	ILMN_12611	0.000840226	3.26
LRIG1	ILMN_3319	0.000196562	3.25
CAV1	ILMN_178269	1.85E-05	3.25
CRISPLD2	ILMN_7921	0.000247317	3.21
CYB561	ILMN_8373	3.57E-05	3.16
H2AFY	ILMN_2037	4.03E-05	3.13
ANTXR1	ILMN_14947	2.10E-05	3.12
PDGFRB	ILMN_25767	0.000268609	3.08
RRAS2	ILMN_165732	0.000660422	2.99
BIN1	ILMN_22961	0.00088286	2.99
ECOP	ILMN_30115	0.000103824	2.98
CD151	ILMN_44972	2.45E-05	2.97
LOC728855	ILMN_371939	0.000360861	2.97
LRIG1	ILMN_3319	0.000108921	2.95
PLEC1	ILMN_27233	0.000244607	2.94
UBA7	ILMN_22067	0.000155014	2.92
FHL1	ILMN_7975	0.000447745	2.91
ELL2	ILMN_15317	9.79E-05	2.90
SMTN	ILMN_18885	0.000408067	2.89
CYB561	ILMN_20474	1.03E-05	2.88
GBP1	ILMN_28413	0.000178108	2.88
SLC25A24	ILMN_183710	4.78E-05	2.87
CDH13	ILMN_26240	7.04E-05	2.87
DPYSL2	ILMN_9671	1.57E-06	2.87

Symbol	Probe ID	p-value	Fold-Change
NTN4	ILMN_29679	3.45E-05	2.87
CAV2	ILMN_177870	6.30E-05	2.84
COL12A1	ILMN_12229	0.000296527	2.84
EDN1	ILMN_28724	0.000650459	2.82
BDNF	ILMN_7507	0.000238527	2.78
FAM113B	ILMN_9482	0.000745868	2.78
CCDC102A	ILMN_12942	9.96E-05	2.77
STARD13	ILMN_4466	0.000645096	2.77
EGFR	ILMN_164647	5.55E-05	2.77
SCHIP1	ILMN_29529	0.000921965	2.76
C3orf59	ILMN_14619	9.86E-05	2.74
SDC2	ILMN_12108	0.000571732	2.73
BMP1	ILMN_6483	0.000351816	2.72
SLC14A1	ILMN_10720	0.00051494	2.72
PPAP2A	ILMN_5193	3.40E-05	2.72
TBX3	ILMN_23090	0.000270281	2.72
DLX1	ILMN_164629	0.000146461	2.71
ACPL2	ILMN_138667	9.44E-05	2.70
DDAH1	ILMN_182204	0.000707959	2.70
TMEM132A	ILMN_41781	0.000404537	2.70
C21orf7	ILMN_9078	1.22E-05	2.69
TGFBR2	ILMN_22189	4.79E-06	2.69
IL7R	ILMN_10513	9.97E-05	2.67
LOC387841	ILMN_37989	0.000238086	2.66
TGFBR2	ILMN_22189	8.29E-06	2.66
VOPP1	ILMN_30115	3.55E-05	2.65
SEMA4B	ILMN_25258	0.000165652	2.65
TIPARP	ILMN_4419	1.47E-05	2.64
SLC44A2	ILMN_5176	0.000602377	2.63
GLIS3	ILMN_25239	0.000114088	2.63
PAWR	ILMN_18537	0.000365278	2.62
TMEM44	ILMN_165329	0.000311266	2.62
SMOX	ILMN_14770	0.0004041	2.60
SLIT2	ILMN_12361	0.000373011	2.59
ACPL2	ILMN_7605	0.000400906	2.58
EML1	ILMN_8689	0.000797292	2.58
PITX1	ILMN_19276	0.000707844	2.57
TMEM45A	ILMN_30168	6.02E-05	2.57

(*Continued*)

Symbol	Probe ID	p-value	Fold-Change
ENG	ILMN_18184	0.000947904	2.57
BIRC2	ILMN_23760	0.000171375	2.56
EXT1	ILMN_18298	3.49E-05	2.55
GAD1	ILMN_4673	2.05E-05	2.54
STX1A	ILMN_11463	0.000228726	2.54
IRS1	ILMN_18432	0.000818928	2.53
EFEMP1	ILMN_41541	0.000952514	2.52
PTPRE	ILMN_23422	0.000459091	2.52
RUNX2	ILMN_11767	0.000189897	2.52
MGC16121	ILMN_171797	0.000242529	2.51
AHR	ILMN_174431	0.000640662	2.51
SMAD6	ILMN_169773	0.000868132	2.49
VCL	ILMN_26712	0.000882981	2.49
C17orf60	ILMN_307137	0.000363649	2.49
FUT8	ILMN_12324	0.000181522	2.48
SSBP2	ILMN_5320	0.000387408	2.44
NMI	ILMN_3552	3.46E-07	2.43
TRPC1	ILMN_182033	1.71E-05	2.42
WNK4	ILMN_180414	0.000475743	2.41
ZHX3	ILMN_6636	0.000359101	2.40
SLC4A7	ILMN_163925	0.000440965	2.40
LOC100133999	ILMN_347150	0.00018259	2.40
RILPL2	ILMN_2260	0.000361433	2.40
RGS17	ILMN_25210	0.000322783	2.39
CCL5	ILMN_22732	0.000919953	2.39
FAM176B	ILMN_3804	0.000271883	2.37
SPARC	ILMN_1780	0.000261452	2.37
COL13A1	ILMN_507	0.000276227	2.36
ARHGAP22	ILMN_15801	0.000174159	2.36
MEX3B	ILMN_29516	0.000381794	2.35
GOLPH4	ILMN_179486	0.000354709	2.35
ADK	ILMN_4107	0.000123904	2.35
DPYD	ILMN_19002	2.11E-05	2.35
	ILMN_123169	3.06E-05	2.34
OSMR	ILMN_165419	6.22E-05	2.34
UAP1	ILMN_162883	0.000404853	2.32
SMOX	ILMN_14642	0.000458511	2.32
KLF10	ILMN_2466	4.17E-05	2.32

Symbol	Probe ID	p-value	Fold-Change
CDK6	ILMN_178275	0.00034469	2.31
ASAM	ILMN_27333	0.000229548	2.31
PLK2	ILMN_5648	0.000254697	2.31
LOC728069	ILMN_169318	0.000676845	2.28
LOC643319	ILMN_41137	5.63E-05	2.28
GAD1	ILMN_4770	0.000128466	2.28
TMEM44	ILMN_165329	4.73E-05	2.28
LOC653778	ILMN_32201	8.89E-05	2.21
CAMK2G	ILMN_25332	0.000906732	2.21
LOC654103	ILMN_37027	0.00067133	2.20
CDKN2C	ILMN_36950	2.45E-05	2.16
ULBP2	ILMN_27661	0.000222377	2.15
COL13A1	ILMN_25843	0.000337238	2.15
P4HA2	ILMN_9080	0.000670117	2.13
ZNFX1	ILMN_176843	0.000215938	2.12
FN1	ILMN_20090	0.00086466	2.11
TAGLN2	ILMN_14546	1.37E-06	2.11
TRAM2	ILMN_26252	0.000323518	2.11
PLP2	ILMN_21868	0.000914472	2.10
CAPN2	ILMN_24443	0.000837345	2.10
NR2F1	ILMN_177945	0.000670925	2.10
LOC493869	ILMN_7364	0.000538336	2.10
LEPRE1	ILMN_165693	0.000186912	2.09
NFKBIA	ILMN_6745	2.51E-05	2.06
ARSJ	ILMN_24631	3.85E-05	2.05
CDC42EP1	ILMN_9329	0.000185168	2.05
TMEM189-UBE2V1	ILMN_20084	0.000416263	2.03
PDLIM7	ILMN_28275	4.81E-05	2.03
NRIP1	ILMN_4339	6.23E-05	2.01
BIRC2	ILMN_23760	0.000209075	2.01
FYN	ILMN_25662	0.000242352	–2.01
CDC16	ILMN_175130	6.99E-06	–2.02
WDSUB1	ILMN_9810	0.000392264	–2.02
SLC25A13	ILMN_162783	2.62E-06	–2.03
C21orf91	ILMN_19108	0.000133742	–2.03
MYO9B	ILMN_25414	0.000125509	–2.04
STRADB	ILMN_24336	9.93E-05	–2.04

(*Continued*)

Symbol	Probe ID	p-value	Fold-Change
GBA	ILMN_28933	0.000419869	–2.05
SDHC	ILMN_14364	1.06E-05	–2.05
CCDC43	ILMN_306768	0.000204178	–2.05
ST7	ILMN_15870	0.000138737	–2.06
CLCN7	ILMN_8600	1.31E-06	–2.07
C12orf5	ILMN_183781	2.02E-05	–2.07
CHMP1B	ILMN_27331	3.55E-05	–2.07
PPFIA1	ILMN_28981	0.000582279	–2.08
C5orf32	ILMN_11272	0.000517774	–2.08
SLC3A2	ILMN_12826	2.14E-08	–2.08
CHKA	ILMN_28401	1.97E-05	–2.09
CDC16	ILMN_175130	9.49E-07	–2.09
SEC11C	ILMN_29023	2.67E-09	–2.09
RPAP1	ILMN_22842	0.000894721	–2.10
HSPA4	ILMN_166427	0.000926623	–2.12
DUSP22	ILMN_15436	0.000635356	–2.13
EPS15	ILMN_183380	0.000394287	–2.13
IDH3A	ILMN_3303	0.000266058	–2.13
SGK	ILMN_2451	5.27E-05	–2.13
YOD1	ILMN_19081	0.000220719	–2.13
PPFIA1	ILMN_28981	0.000483093	–2.14
SEH1L	ILMN_177006	0.000684094	–2.15
SLC22A23	ILMN_356887	0.000527871	–2.15
GNPTAB	ILMN_5374	0.000511113	–2.15
SCARB1	ILMN_5375	2.84E-05	–2.16
FAHD1	ILMN_2449	6.58E-06	–2.18
PAFAH2	ILMN_3410	0.000189882	–2.19
MRPL44	ILMN_16607	2.50E-05	–2.20
FASTKD1	ILMN_5271	0.000911014	–2.20
SPIRE1	ILMN_2975	3.47E-06	–2.20
NIN	ILMN_172996	0.000375173	–2.21
CHD7	ILMN_29669	0.000765999	–2.22
SAPS1	ILMN_21557	0.000568904	–2.22
ZNF654	ILMN_6649	0.000215631	–2.22
ZEB2	ILMN_14685	0.000334629	–2.22
FBXO2	ILMN_166168	0.000649004	–2.24

Symbol	Probe ID	p-value	Fold-Change
TMEM199	ILMN_3696	7.18E-05	–2.24
NAB2	ILMN_17333	2.26E-05	–2.24
BACE2	ILMN_12258	0.000914343	–2.26
ATP6V0A2	ILMN_23163	0.000106488	–2.26
TOMM40L	ILMN_42128	0.000414352	–2.27
SGK1	ILMN_2451	6.27E-05	–2.27
LOC730167	ILMN_342323	0.000269087	–2.28
TBC1D14	ILMN_165668	8.99E-05	–2.29
MAP3K11	ILMN_23190	9.83E-05	–2.29
SLC31A1	ILMN_28027	4.76E-07	–2.29
RETSAT	ILMN_28492	0.00037606	–2.29
TMEM170B	ILMN_339531	0.00050239	–2.31
C12orf49	ILMN_20444	0.00034345	–2.31
CDC16	ILMN_28112	1.36E-06	–2.32
MARS2	ILMN_19018	4.25E-06	–2.33
VAT1	ILMN_26285	0.000163	–2.33
SLC25A4	ILMN_2485	6.11E-06	–2.33
EXOC2	ILMN_172388	6.35E-05	–2.34
GGA2	ILMN_17168	0.000621985	–2.34
SNX30	ILMN_309807	0.000701473	–2.34
SGK1	ILMN_2451	9.43E-05	–2.35
USP48	ILMN_163754	6.74E-06	–2.35
STXBP1	ILMN_182537	0.000967824	–2.35
ZNF689	ILMN_14716	0.00028809	–2.35
ZBTB24	ILMN_6521	5.61E-07	–2.36
EXOC2	ILMN_172388	0.000313823	–2.37
ACSL3	ILMN_416	0.000286463	–2.37
DUSP22	ILMN_15436	6.40E-05	–2.37
FCGR2B	ILMN_27581	0.000272575	–2.37
NAP1L5	ILMN_5355	0.000456444	–2.38
FABP5L2	ILMN_177732	0.000172346	–2.38
HAGH	ILMN_22401	5.84E-05	–2.38
C17orf63	ILMN_173887	1.66E-07	–2.41
FAM195A	ILMN_9509	0.000226578	–2.41
GAS8	ILMN_26809	0.000102631	–2.41
DAAM1	ILMN_183695	0.00060562	–2.41

(*Continued*)

Symbol	Probe ID	p-value	Fold-Change
IVNS1ABP	ILMN_26781	4.19E-05	–2.43
MESDC1	ILMN_18570	9.25E-05	–2.43
MYCBP2	ILMN_7642	0.000432138	–2.44
C15orf39	ILMN_18382	0.000237453	–2.45
SLC43A3	ILMN_165230	0.000456408	–2.45
ME2	ILMN_176679	3.47E-05	–2.46
FLJ38482	ILMN_2215	1.89E-05	–2.47
FABP5	ILMN_27564	0.000340688	–2.48
VEGFB	ILMN_15862	0.000351163	–2.49
ZNF697	ILMN_174344	0.000130113	–2.50
SLAIN1	ILMN_165142	0.00039226	–2.50
MSI2	ILMN_525	3.22E-05	–2.52
PIK3CB	ILMN_22394	0.000190395	–2.52
LOC731007	ILMN_176314	0.00064071	–2.54
FASTKD2	ILMN_12269	0.000125027	–2.54
AGA	ILMN_165746	0.000253853	–2.54
UBL3	ILMN_25010	2.88E-05	–2.55
ABHD6	ILMN_9952	1.25E-05	–2.55
HS1BP3	ILMN_1874	0.000126476	–2.56
THNSL1	ILMN_10160	7.16E-05	–2.56
IVNS1ABP	ILMN_26781	6.20E-07	–2.56
ZNF280B	ILMN_19056	0.000124126	–2.57
ZADH2	ILMN_5633	0.000249744	–2.58
EN2	ILMN_171364	0.000374559	–2.58
STX3	ILMN_18859	1.17E-05	–2.59
SLC39A6	ILMN_170037	2.08E-07	–2.60
IDI1	ILMN_20349	0.000707879	–2.60
CYBASC3	ILMN_28843	0.000131069	–2.60
USP48	ILMN_29650	0.000120015	–2.63
ANKRD28	ILMN_161875	8.31E-05	–2.63
PRR5	ILMN_5791	0.000480657	–2.63
LOC654244	ILMN_31329	1.22E-05	–2.66
BAMBI	ILMN_8469	0.000107735	–2.66
VAC14	ILMN_30132	1.34E-05	–2.68
PLEKHG3	ILMN_28109	4.25E-05	–2.69
TMEM192	ILMN_307671	2.57E-06	–2.69

Symbol	Probe ID	p-value	Fold-Change
NARS2	ILMN_13605	1.48E-05	–2.71
PAG1	ILMN_20316	0.000791062	–2.72
C14orf109	ILMN_307789	1.99E-08	–2.72
CHKA	ILMN_28401	1.52E-05	–2.73
MIPEP	ILMN_24370	5.99E-05	–2.75
QPRT	ILMN_21680	0.000323818	–2.76
CDS2	ILMN_18323	1.11E-06	–2.76
PTPRM	ILMN_19957	8.59E-05	–2.77
PLD1	ILMN_20063	9.36E-05	–2.79
SYNGR1	ILMN_20235	0.000850295	–2.80
STOM	ILMN_183628	5.58E-05	–2.82
KIAA1598	ILMN_4741	0.000208839	–2.82
LOC440731	ILMN_36463	5.89E-06	–2.83
ACSL1	ILMN_12367	0.000430032	–2.84
LOC729009	ILMN_347545	0.000950114	–2.85
	ILMN_91344	0.000357348	–2.87
FTHL3	ILMN_27691	0.000637655	–2.87
RPS6KA5	ILMN_13156	0.000311936	–2.88
AVPI1	ILMN_9920	7.69E-07	–2.88
SLC25A4	ILMN_2485	2.10E-05	–2.88
SMPDL3A	ILMN_16204	0.000492497	–2.88
RANBP10	ILMN_21091	3.43E-06	–2.89
MGC13057	ILMN_13440	0.000455491	–2.90
POLR3G	ILMN_13297	8.38E-06	–2.91
SLC7A5	ILMN_25446	6.96E-07	–2.91
PSEN2	ILMN_4951	4.25E-06	–2.93
P2RX4	ILMN_25537	6.31E-05	–2.93
SLC3A2	ILMN_12826	0.00014587	–2.94
AMDHD2	ILMN_26534	5.52E-05	–2.95
CPEB1	ILMN_183191	1.60E-05	–2.96
C14orf109	ILMN_307789	3.16E-07	–2.96
BCL2	ILMN_171007	3.49E-05	–2.96
PAG1	ILMN_174074	0.000391055	–2.96
MOAP1	ILMN_165500	8.20E-07	–2.97
LOC389599	ILMN_165271	6.45E-05	–2.98
HAGHL	ILMN_15715	0.000154931	–2.99

(*Continued*)

Symbol	Probe ID	p-value	Fold-Change
RTN4R	ILMN_13643	5.03E-05	–2.99
IRF4	ILMN_12414	0.000123787	–2.99
DSTYK	ILMN_370446	0.000175241	–2.99
FIG4	ILMN_20404	3.94E-05	–3.01
ABCC2	ILMN_9691	0.00054703	–3.01
ENOSF1	ILMN_12398	9.77E-06	–3.01
TGFBRAP1	ILMN_30176	0.000126304	–3.01
FAM53B	ILMN_26314	0.000969088	–3.01
MYO10	ILMN_28857	3.80E-05	–3.02
SLC11A2	ILMN_10129	6.52E-05	–3.03
METTL9	ILMN_22037	0.000183645	–3.04
RNF144B	ILMN_9298	0.00013559	–3.05
STXBP6	ILMN_18387	0.000614655	–3.05
WDR91	ILMN_20547	0.000197012	–3.07
SLC16A10	ILMN_10556	4.31E-05	–3.08
CPEB2	ILMN_3402	0.000338478	–3.11
NUP98	ILMN_21954	1.51E-06	–3.13
LOC284988	ILMN_164134	0.000167525	–3.13
QDPR	ILMN_26096	2.75E-05	–3.15
ATP6V1C1	ILMN_181099	8.56E-05	–3.17
	ILMN_70822	0.000385505	–3.17
RAB27A	ILMN_26005	1.53E-05	–3.18
LOC645904	ILMN_39266	2.60E-06	–3.18
FLJ46906	ILMN_40537	0.00015516	–3.22
POLR3G	ILMN_13297	1.82E-05	–3.22
PTP4A1	ILMN_165831	2.21E-05	–3.24
STXBP1	ILMN_10266	0.00011764	–3.27
GYPC	ILMN_17566	2.07E-06	–3.28
KCNN2	ILMN_165586	0.000389758	–3.30
TPD52	ILMN_8812	1.78E-05	–3.31
GYPC	ILMN_17566	3.06E-06	–3.33
LOC644590	ILMN_45611	2.68E-05	–3.33
MYO5A	ILMN_12618	0.000114869	–3.35
SLC22A18	ILMN_20563	9.14E-05	–3.36
CPEB1	ILMN_183191	1.10E-06	–3.36
STOM	ILMN_183628	8.43E-05	–3.41

Symbol	Probe ID	p-value	Fold-Change
DCLK1	ILMN_178525	0.000737489	–3.41
C22orf25	ILMN_13683	8.76E-05	–3.43
MAD1L1	ILMN_9328	4.22E-08	–3.45
DSTYK	ILMN_370437	0.00014763	–3.46
ASAH1	ILMN_26236	7.73E-06	–3.46
C19orf28	ILMN_11361	0.000170076	–3.46
LOC642956	ILMN_42416	0.000303075	–3.46
CDK2	ILMN_12332	2.80E-11	–3.48
HPS4	ILMN_7734	3.41E-05	–3.50
SETDB2	ILMN_24431	7.55E-07	–3.52
OSTM1	ILMN_21496	4.18E-05	–3.52
TBC1D7	ILMN_10292	1.74E-08	–3.53
TDRD7	ILMN_27692	1.95E-05	–3.54
STXBP6	ILMN_18387	0.000187123	–3.54
LOC388588	ILMN_33026	4.85E-05	–3.55
NPL	ILMN_25291	0.00018053	–3.56
PIK3CD	ILMN_15406	0.00018874	–3.59
RIPK5	ILMN_27483	0.000119582	–3.61
C9orf91	ILMN_21211	2.95E-06	–3.61
GREB1	ILMN_167773	3.93E-05	–3.62
C5orf22	ILMN_19584	1.07E-06	–3.67
BCL2	ILMN_171007	8.44E-06	–3.68
CDH3	ILMN_12740	0.000601941	–3.74
SOX13	ILMN_20138	3.11E-07	–3.78
PROS1	ILMN_3398	0.000374802	–3.82
FABP5L2	ILMN_336422	0.000156471	–3.84
DYNC1I1	ILMN_7312	0.000334358	–3.85
STX7	ILMN_23974	3.75E-06	–3.88
NPL	ILMN_25291	5.96E-05	–3.89
PIR	ILMN_13999	2.77E-05	–3.90
C10orf11	ILMN_17394	2.72E-05	–3.91
GPR137B	ILMN_10711	2.23E-05	–3.92
GK	ILMN_10128	1.07E-05	–3.94
TMEM8	ILMN_19628	8.19E-08	–3.97
SNCA	ILMN_2235	1.28E-06	–4.04
SYNGR1	ILMN_175984	7.29E-05	–4.05

(Continued)

Symbol	Probe ID	p-value	Fold-Change
C6orf192	ILMN_166002	0.000362297	–4.06
INPP4B	ILMN_22637	4.55E-07	–4.07
NEDD4L	ILMN_18912	1.50E-07	–4.10
PRDM7	ILMN_307242	0.000326591	–4.12
BIRC7	ILMN_21032	2.48E-05	–4.13
KU-MEL-3	ILMN_3790	0.000212321	–4.15
TBC1D16	ILMN_17194	4.98E-07	–4.16
OR7E156P	ILMN_13738	3.45E-05	–4.18
SLC39A6	ILMN_170037	8.04E-07	–4.18
	ILMN_84306	0.000224537	–4.19
SORT1	ILMN_165748	6.32E-06	–4.26
PSEN2	ILMN_21140	1.10E-06	–4.30
DLL3	ILMN_21363	0.00041846	–4.34
GPM6B	ILMN_23991	0.000574913	–4.34
SIRPA	ILMN_166179	1.87E-05	–4.36
FAM174B	ILMN_1130	9.87E-05	–4.37
PPM1H	ILMN_308090	2.41E-05	–4.39
SLC3A2	ILMN_178961	4.51E-09	–4.39
TNFRSF14	ILMN_3329	2.37E-06	–4.41
HRASLS3	ILMN_15631	0.000843533	–4.44
EPB41L3	ILMN_14465	0.000108819	–4.44
CYFIP2	ILMN_15047	0.00011641	–4.49
LZTS1	ILMN_3351	0.000121107	–4.50
PIR	ILMN_13999	9.22E-06	–4.54
GK	ILMN_28282	2.24E-06	–4.57
CEACAM1	ILMN_21651	0.000215908	–4.57
GPM6B	ILMN_23991	0.000540196	–4.65
LAMA1	ILMN_27530	1.66E-08	–4.66
SOX10	ILMN_13184	0.000646769	–4.68
KIAA1026	ILMN_3555	3.80E-08	–4.70
RUNX3	ILMN_166428	6.26E-05	–4.71
RENBP	ILMN_17151	0.000132876	–4.74
ST6GALNAC2	ILMN_15305	0.000294835	–4.80
ACP5	ILMN_20083	0.000242815	–4.81
FAM69B	ILMN_173053	3.06E-05	–4.84
SLC7A8	ILMN_22403	4.48E-05	–4.92

Symbol	Probe ID	p-value	Fold-Change
GJB1	ILMN_8143	1.43E-05	–5.02
GPR19	ILMN_20675	8.22E-08	–5.08
GPR143	ILMN_15627	0.000393721	–5.18
SLC45A2	ILMN_16585	6.28E-06	–5.24
ZFYVE16	ILMN_25838	2.43E-07	–5.24
PARD6G	ILMN_4804	1.14E-09	–5.28
LONRF1	ILMN_38387	2.68E-07	–5.29
PPFIBP2	ILMN_183115	1.98E-06	–5.34
MICAL1	ILMN_18994	3.33E-06	–5.43
SNCA	ILMN_3516	3.20E-07	–5.43
MC1R	ILMN_25536	9.39E-07	–5.54
LOC440459	ILMN_183612	0.00017076	–5.55
SLC1A4	ILMN_12585	2.08E-05	–5.59
KIT	ILMN_306843	0.000332358	–5.65
TIMP2	ILMN_166675	1.06E-06	–5.66
RGS12	ILMN_161894	1.10E-05	–5.71
LGALS3	ILMN_14333	5.94E-06	–5.75
CEACAM1	ILMN_20142	9.39E-05	–5.85
CTSH	ILMN_8602	3.76E-08	–5.95
FAIM3	ILMN_28190	1.14E-05	–5.96
C19orf28	ILMN_161980	1.20E-05	–5.98
C6orf218	ILMN_27124	9.17E-09	–6.16
RRAGD	ILMN_5663	1.98E-06	–6.19
SIRPA	ILMN_1639	2.10E-07	–6.25
RAP1GAP	ILMN_13405	2.93E-06	–6.27
MYO1D	ILMN_22644	2.47E-07	–6.47
CTSH	ILMN_8602	6.86E-07	–6.61
HES6	ILMN_4854	5.37E-08	–6.69
CDK5R1	ILMN_4868	4.34E-09	–6.77
LOC387763	ILMN_43061	3.51E-05	–6.80
CABLES1	ILMN_26976	2.69E-08	–6.84
EDNRB	ILMN_22613	4.58E-05	–6.93
CDK2	ILMN_12332	1.17E-08	–7.01
TUBB4	ILMN_23388	2.31E-06	–7.07
TTYH2	ILMN_25738	2.45E-07	–7.11
ST3GAL6	ILMN_2870	1.09E-06	–7.41

(*Continued*)

Symbol	Probe ID	p-value	Fold-Change
RGS1	ILMN_177208	0.000198809	–7.63
BEST1	ILMN_19773	8.00E-07	–7.66
ADCY1	ILMN_179982	5.65E-06	–7.69
SLC16A6	ILMN_14509	0.000232387	–7.70
CEACAM1	ILMN_20142	8.83E-06	–8.19
ITPKB	ILMN_12468	1.35E-07	–8.31
IGSF11	ILMN_551	7.09E-08	–8.39
TMC6	ILMN_19035	8.31E-08	–8.44
MBP	ILMN_14913	8.43E-08	–8.61
QPCT	ILMN_6510	1.75E-05	–9.37
CA14	ILMN_2282	1.86E-06	–9.39
RAB38	ILMN_22951	5.85E-06	–9.50
CHCHD6	ILMN_19685	1.36E-09	–9.51
PHACTR1	ILMN_17425	3.05E-07	–9.86
MITF	ILMN_1045	1.09E-07	–9.87
TRIM63	ILMN_27419	2.80E-09	–10.05
GDF15	ILMN_2688	0.00058484	–10.34
MBP	ILMN_10370	1.35E-09	–10.43
MBP	ILMN_3041	2.37E-08	–10.43
DNAJA4	ILMN_23222	1.21E-05	–10.51
GYG2	ILMN_24869	5.62E-10	–10.58
D4S234E	ILMN_173747	1.30E-06	–11.26
OCA2	ILMN_177862	4.25E-06	–11.56
APOE	ILMN_11525	3.41E-06	–11.66
SLC27A3	ILMN_820	5.04E-06	–11.84
VGF	ILMN_9112	5.85E-06	–12.06
GYG2	ILMN_176856	5.12E-09	–12.30
IGFBP1	ILMN_19001	2.58E-11	–12.31
DCT	ILMN_1796	3.83E-05	–12.57
SEMA6A	ILMN_11282	1.16E-07	–12.60
BIRC7	ILMN_21032	2.10E-07	–13.09
RAB17	ILMN_9806	1.68E-09	–13.19
CDH1	ILMN_29401	1.07E-07	–13.51
LOC641738	ILMN_31380	2.11E-08	–14.15
TSPAN10	ILMN_12531	1.88E-07	–14.39

Symbol	Probe ID	p-value	Fold-Change
SLC45A2	ILMN_29672	5.73E-09	–15.52
CAPN3	ILMN_8100	1.11E-07	–15.93
APOLD1	ILMN_21263	1.89E-10	–16.67
LAMA1	ILMN_27530	1.42E-11	–17.05
CAPN3	ILMN_25522	9.31E-08	–17.61
SLC45A2	ILMN_29672	4.55E-10	–22.25
SLC45A2	ILMN_29672	9.68E-10	–25.05
TYR	ILMN_16044	5.94E-08	–27.25
MLANA	ILMN_22830	2.27E-09	–36.94
SILV	ILMN_9512	1.23E-10	–75.07

Supplementary Table S2.

Mesenchymal-phenotype enrichment			
**Gene Set**	**NES**	**NOM p-val**	**FDR q-val**
**follow link to MSigDB**			
CHARAFE_BREAST_CANCER_LUMINAL_VS_MESENCHYMAL_DN	1.87	0	0
SCHUETZ_BREAST_CANCER_DUCTAL_INVASIVE_UP	1.87	0	0
VECCHI_GASTRIC_CANCER_ADVANCED_VS_EARLY_UP	1.86	0	0
REN_ALVEOLAR_RHABDOMYOSARCOMA_DN	1.86	0	0
REACTOME_EXTRACELLULAR_MATRIX_ORGANIZATION	1.86	0	0
REACTOME_COLLAGEN_FORMATION	1.85	0	0
ANASTASSIOU_CANCER_MESENCHYMAL_TRANSITION_SIGNATURE	1.83	0	0
KIM_GLIS2_TARGETS_UP	1.82	0	0
PETROVA_ENDOTHELIUM_LYMPHATIC_VS_BLOOD_DN	1.82	0	0
WIEDERSCHAIN_TARGETS_OF_BMI1_AND_PCGF2	1.81	0	0
ONDER_CDH1_TARGETS_2_UP	1.81	0	0
GU_PDEF_TARGETS_UP	1.8	0	0
KEGG_GRAFT_VERSUS_HOST_DISEASE	1.79	0	0.001
VART_KSHV_INFECTION_ANGIOGENIC_MARKERS_UP	1.78	0	0.001
WANG_SMARCE1_TARGETS_UP	1.78	0	0.001
POTTI_TOPOTECAN_SENSITIVITY	1.77	0	0.001
PID_INTEGRIN3_PATHWAY	1.77	0	0.001
PHONG_TNF_RESPONSE_VIA_P38_PARTIAL	1.77	0	0.001
PID_INTEGRIN1_PATHWAY	1.77	0.002	0.001
PASINI_SUZ12_TARGETS_DN	1.76	0	0.001
AZARE_NEOPLASTIC_TRANSFORMATION_BY_STAT3_DN	1.75	0	0.002
CHARAFE_BREAST_CANCER_LUMINAL_VS_BASAL_DN	1.74	0	0.002
HINATA_NFKB_TARGETS_KERATINOCYTE_UP	1.74	0.002	0.002
GRAHAM_CML_DIVIDING_VS_NORMAL_QUIESCENT_DN	1.73	0	0.003
GAURNIER_PSMD4_TARGETS	1.73	0	0.003
CROMER_TUMORIGENESIS_UP	1.72	0	0.004
WESTON_VEGFA_TARGETS_6HR	1.72	0	0.004
WILCOX_PRESPONSE_TO_ROGESTERONE_DN	1.72	0	0.004
PICCALUGA_ANGIOIMMUNOBLASTIC_LYMPHOMA_UP	1.72	0	0.004
SANA_TNF_SIGNALING_UP	1.71	0	0.004
ZHU_CMV_24_HR_DN	1.71	0	0.004
CHEN_LVAD_SUPPORT_OF_FAILING_HEART_UP	1.71	0	0.004
PID_AVB3_INTEGRIN_PATHWAY	1.71	0	0.005
SWEET_KRAS_TARGETS_UP	1.71	0	0.006

Mesenchymal-phenotype enrichment			
**Gene Set**	**NES**	**NOM p-val**	**FDR q-val**
**follow link to MSigDB**			
BOQUEST_STEM_CELL_UP	1.7	0	0.006
JECHLINGER_EPITHELIAL_TO_MESENCHYMAL_TRANSITION_UP	1.7	0	0.006
VERRECCHIA_EARLY_RESPONSE_TO_TGFB1	1.7	0	0.006
ZWANG_CLASS_2_TRANSIENTLY_INDUCED_BY_EGF	1.7	0	0.006
KEGG_TYPE_I_DIABETES_MELLITUS	1.69	0	0.007
KEGG_ECM_RECEPTOR_INTERACTION	1.69	0	0.006
PHONG_TNF_RESPONSE_NOT_VIA_P38	1.69	0	0.007
KEGG_AUTOIMMUNE_THYROID_DISEASE	1.69	0	0.007
BRUECKNER_TARGETS_OF_MIRLET7A3_DN	1.69	0	0.007
VART_KSHV_INFECTION_ANGIOGENIC_MARKERS_DN	1.69	0	0.007
PETROVA_PROX1_TARGETS_DN	1.69	0	0.007
WU_CELL_MIGRATION	1.69	0	0.007
KEGG_HYPERTROPHIC_CARDIOMYOPATHY_HCM	1.68	0	0.007
KEGG_ASTHMA	1.68	0	0.008
HUANG_DASATINIB_RESISTANCE_UP	1.68	0	0.008
SASAI_RESISTANCE_TO_NEOPLASTIC_TRANSFROMATION	1.68	0	0.008
SENESE_HDAC1_AND_HDAC2_TARGETS_DN	1.68	0	0.008
SCHOEN_NFKB_SIGNALING	1.68	0	0.008
MIYAGAWA_TARGETS_OF_EWSR1_ETS_FUSIONS_DN	1.68	0	0.008
BASAKI_YBX1_TARGETS_UP	1.67	0	0.009
ZHU_CMV_ALL_DN	1.67	0	0.009
REACTOME_INTERFERON_ALPHA_BETA_SIGNALING	1.67	0	0.009
GRAESSMANN_RESPONSE_TO_MC_AND_SERUM_DEPRIVATION_UP	1.67	0	0.009
SENESE_HDAC2_TARGETS_DN	1.67	0	0.01
ONDER_CDH1_TARGETS_3_DN	1.66	0	0.01
ALTEMEIER_RESPONSE_TO_LPS_WITH_MECHANICAL_VENTILATION	1.66	0	0.01
CROONQUIST_NRAS_VS_STROMAL_STIMULATION_DN	1.66	0	0.011
LIM_MAMMARY_STEM_CELL_UP	1.66	0	0.011
DASU_IL6_SIGNALING_UP	1.66	0.002	0.011
RODWELL_AGING_KIDNEY_NO_BLOOD_UP	1.66	0	0.012
PID_SYNDECAN_1_PATHWAY	1.66	0	0.012
RIGGI_EWING_SARCOMA_PROGENITOR_DN	1.65	0	0.013
SWEET_LUNG_CANCER_KRAS_DN	1.65	0	0.014
NAKAMURA_TUMOR_ZONE_PERIPHERAL_VS_CENTRAL_UP	1.65	0	0.014
TSAI_RESPONSE_TO_RADIATION_THERAPY	1.65	0	0.014

(*Continued*)

Mesenchymal-phenotype enrichment			
**Gene Set**	**NES**	**NOM p-val**	**FDR q-val**
**follow link to MSigDB**			
SEITZ_NEOPLASTIC_TRANSFORMATION_BY_8P_DELETION_UP	1.65	0	0.014
RODWELL_AGING_KIDNEY_UP	1.65	0	0.014
KEGG_ALLOGRAFT_REJECTION	1.65	0.002	0.014
REACTOME_NCAM1_INTERACTIONS	1.64	0	0.016
REACTOME_INTERFERON_GAMMA_SIGNALING	1.64	0	0.016
BIOCARTA_INFLAM_PATHWAY	1.64	0	0.016
GOTZMANN_EPITHELIAL_TO_MESENCHYMAL_TRANSITION_UP	1.64	0.002	0.017
CLASPER_LYMPHATIC_VESSELS_DURING_METASTASIS_DN	1.63	0	0.019
MURATA_VIRULENCE_OF_H_PILORI	1.63	0	0.019
CHARAFE_BREAST_CANCER_BASAL_VS_MESENCHYMAL_DN	1.63	0.002	0.019
HECKER_IFNB1_TARGETS	1.63	0	0.019
ZHANG_ANTIVIRAL_RESPONSE_TO_RIBAVIRIN_UP	1.63	0.002	0.019
WINZEN_DEGRADED_VIA_KHSRP	1.63	0.002	0.019
LINDGREN_BLADDER_CANCER_CLUSTER_2B	1.63	0	0.02
YAO_TEMPORAL_RESPONSE_TO_PROGESTERONE_CLUSTER_16	1.63	0	0.02
PHONG_TNF_TARGETS_UP	1.62	0.002	0.021
LIU_TARGETS_OF_VMYB_VS_CMYB_DN	1.62	0.002	0.02
BURTON_ADIPOGENESIS_PEAK_AT_2HR	1.62	0	0.02
ONDER_CDH1_SIGNALING_VIA_CTNNB1	1.62	0	0.022
BEGUM_TARGETS_OF_PAX3_FOXO1_FUSION_DN	1.62	0	0.021
YAMASHITA_METHYLATED_IN_PROSTATE_CANCER	1.62	0	0.021
CHIBA_RESPONSE_TO_TSA_UP	1.62	0.004	0.022
DER_IFN_BETA_RESPONSE_UP	1.62	0	0.022
TURASHVILI_BREAST_LOBULAR_CARCINOMA_VS_DUCTAL_NORMAL_UP	1.62	0	0.022
GENTILE_UV_HIGH_DOSE_DN	1.62	0	0.023
CROONQUIST_STROMAL_STIMULATION_UP	1.61	0.002	0.024
IGLESIAS_E2F_TARGETS_UP	1.61	0	0.024
BILD_HRAS_ONCOGENIC_SIGNATURE	1.61	0	0.023
CHIARADONNA_NEOPLASTIC_TRANSFORMATION_CDC25_UP	1.61	0	0.024
WEINMANN_ADAPTATION_TO_HYPOXIA_DN	1.61	0.002	0.024
DAVICIONI_TARGETS_OF_PAX_FOXO1_FUSIONS_UP	1.61	0	0.024
ICHIBA_GRAFT_VERSUS_HOST_DISEASE_D7_UP	1.61	0	0.025
RICKMAN_HEAD_AND_NECK_CANCER_C	1.61	0.002	0.026
KEGG_FOCAL_ADHESION	1.61	0	0.026
PID_AP1_PATHWAY	1.61	0.002	0.027

Mesenchymal-phenotype enrichment			
**Gene Set**	**NES**	**NOM p-val**	**FDR q-val**
**follow link to MSigDB**			
ROZANOV_MMP14_TARGETS_UP	1.61	0	0.027
BROWNE_HCMV_INFECTION_18HR_DN	1.6	0	0.028
WESTON_VEGFA_TARGETS_12HR	1.6	0.006	0.028
WONG_ENDMETRIUM_CANCER_DN	1.6	0	0.029
REACTOME_INTERFERON_SIGNALING	1.6	0	0.031
RUIZ_TNC_TARGETS_DN	1.6	0	0.031
HINATA_NFKB_TARGETS_FIBROBLAST_UP	1.6	0.002	0.031
GALINDO_IMMUNE_RESPONSE_TO_ENTEROTOXIN	1.6	0	0.031
AMIT_EGF_RESPONSE_240_HELA	1.59	0.004	0.034
SMID_BREAST_CANCER_LUMINAL_A_UP	1.59	0	0.034
REACTOME_STRIATED_MUSCLE_CONTRACTION	1.59	0	0.036
BERTUCCI_MEDULLARY_VS_DUCTAL_BREAST_CANCER_DN	1.59	0	0.037
LAIHO_COLORECTAL_CANCER_SERRATED_UP	1.59	0	0.038
HARRIS_BRAIN_CANCER_PROGENITORS	1.58	0	0.042
DAVICIONI_MOLECULAR_ARMS_VS_ERMS_DN	1.58	0	0.041
HUPER_BREAST_BASAL_VS_LUMINAL_DN	1.58	0.002	0.041
REACTOME_HS_GAG_BIOSYNTHESIS	1.58	0.002	0.042
SUNG_METASTASIS_STROMA_UP	1.58	0	0.043
HOSHIDA_LIVER_CANCER_SUBCLASS_S1	1.58	0	0.045
KEGG_CYTOKINE_CYTOKINE_RECEPTOR_INTERACTION	1.57	0	0.045
VERHAAK_GLIOBLASTOMA_NEURAL	1.57	0	0.045
BURTON_ADIPOGENESIS_3	1.57	0.002	0.045
KEEN_RESPONSE_TO_ROSIGLITAZONE_DN	1.57	0	0.046
KEGG_ARRHYTHMOGENIC_RIGHT_VENTRICULAR_CARDIOMYOPATHY_ARVC	1.57	0.002	0.047
TURASHVILI_BREAST_LOBULAR_CARCINOMA_VS_LOBULAR_NORMAL_DN	1.57	0.006	0.05
KOKKINAKIS_METHIONINE_DEPRIVATION_96HR_UP	1.57	0	0.05
REACTOME_MUSCLE_CONTRACTION	1.57	0.008	0.05

Epithelial-phenotype enrichment			
**Gene Set**	**NES**	**NOM p-val**	**FDR q-val**
**follow link to MSigDB**			
KEGG_OXIDATIVE_PHOSPHORYLATION	–1.76	0	0.011
KEGG_LYSOSOME	–1.7	0	0.036
REACTOME_TCA_CYCLE_AND_RESPIRATORY_ELECTRON_TRANSPORT	–1.7	0	0.05

(*Continued*)

Supplementary Table S3.

Symbol	ProbeID	FC:(class1/class2)	pfp	
PAPSS2	1690259	4.323389537	0	Hs.524491
LUM	2490364	3.486750349	0.005	Hs.406475
IGFBP5	2120524	3.285151117	0.01	Hs.635441
DCN	7650296	3.272251309	0.0233	Hs.156316
IGFBP5	2190674	2.996703626	0.01	Hs.635441
HLA-B	5310168	2.973535534	0.0067	Hs.77961
TGFBI	7650358	2.933411558	0.0056	Hs.369397
PAPPA	730754	2.930832356	0.0067	Hs.643599
HS.552871	1690575	2.765486726	0.006	**HS.552871**
COL5A1	4920369	2.718129927	0.0071	Hs.210283
EEF1A2	6370356	2.664535039	0.0223	Hs.433839
THBS1	5810685	2.66028199	0.0062	Hs.164226
MATN2	5810746	2.63227165	0.0164	Hs.189445
PLA2G4C	1990672	2.491280518	0.035	Hs.631562
HLA-DPA1	1190039	2.365744026	0.0427	Hs.347270
HLA-DMA	540563	2.361275089	0.0438	Hs.351279
HS.562219	5960086	–2.0966	0.031	**HS.562219**
SNORD15B	2710719	–2.243	0.0469	Hs.689080
RPPH1	2260309	–2.37	0.03	Hs.613102
HS.171009	3450379	–2.3845	0.0471	**HS.171009**
IL24	4290201	–2.4175	0.0256	Hs.58831
UBA2	4290632	–2.4322	0.05	Hs.631580
LOC199800	5960735	–2.4582	0.03	Hs.311193
SNORD34	3780050	–2.4926	0.036	
HPDL	6480026	–2.5799	0.0262	Hs.162717
ADRB2	7570326	–2.6116	0.0436	Hs.591251
HS.543887	6840477	–2.7403	0.0075	**HS.543887**
CYORF15A	6860102	–3.528	0.005	Hs.522863
EIF1AY	4150600	–4.4136	0.01	Hs.461178
RPS4Y1	6100687	–5.4352	0.01	Hs.282376
Probe Set ID	Gene Symbol			
201107_s_at	THBS1			
201108_s_at	THBS1			
201109_s_at	THBS1			
201110_s_at	THBS1			
201177_s_at	UBA2			

Symbol	ProbeID	FC:(class1/class2)	pfp	
201506_at	TGFBI			
201744_s_at	LUM			
201893_x_at	DCN			
201909_at	RPS4Y1			
201981_at	PAPPA			
201982_s_at	PAPPA			
202350_s_at	MATN2			
203058_s_at	PAPSS2			
203059_s_at	PAPSS2			
203060_s_at	PAPSS2			
203325_s_at	COL5A1			
204409_s_at	EIF1AY			
204410_at	EIF1AY			
204540_at	EEF1A2			
206569_at	IL24			
209335_at	DCN			
209785_s_at	PLA2G4C			
211813_x_at	DCN			
211896_s_at	DCN			
211990_at	HLA-DPA1			
211991_s_at	HLA-DPA1			
212488_at	COL5A1			
212489_at	COL5A1			
213537_at	HLA-DPA1			
217478_s_at	HLA-DMA			

Supplementary Table S4.

DiI Bright Enrichment			
**GS**	**NES**	**NOM p-val**	**FDR q-val**
**follow link to MSigDB**			
Genes up-regulated in mesenchymal-like melanoma cell line vs epithelial-like cell lines (Supplementary Table S1, current study)	−2.71	0	0
GU_PDEF_TARGETS_UP	−2.52	0	0
DAUER_STAT3_TARGETS_DN	−2.44	0	0
SENESE_HDAC1_AND_HDAC2_TARGETS_DN	−2.42	0	0
SENESE_HDAC2_TARGETS_DN	−2.41	0	0
PID_INTEGRIN1_PATHWAY	−2.38	0	0
SEITZ_NEOPLASTIC_TRANSFORMATION_BY_8P_DELETION_UP	−2.35	0	0
WANG_SMARCE1_TARGETS_UP	−2.35	0	0
BOWIE_RESPONSE_TO_TAMOXIFEN	−2.34	0	0
ANASTASSIOU_CANCER_MESENCHYMAL_TRANSITION_SIGNATURE	−2.34	0	0
TAKEDA_TARGETS_OF_NUP98_HOXA9_FUSION_3D_UP	−2.34	0	0
STAMBOLSKY_TARGETS_OF_MUTATED_TP53_DN	−2.34	0	0
SIMBULAN_PARP1_TARGETS_UP	−2.33	0	0
KIM_GLIS2_TARGETS_UP	−2.33	0	0
GRAHAM_CML_QUIESCENT_VS_NORMAL_QUIESCENT_DN	−2.31	0	0
ZHU_CMV_24_HR_DN	−2.3	0	0
SWEET_KRAS_TARGETS_UP	−2.29	0	0
MOSERLE_IFNA_RESPONSE	−2.28	0	0
GRAHAM_CML_DIVIDING_VS_NORMAL_QUIESCENT_DN	−2.27	0	0

DiI Bright Enrichment			
**GS**	**NES**	**NOM p-val**	**FDR q-val**
**follow link to MSigDB**			
TAKEDA_TARGETS_OF_NUP98_HOXA9_FUSION_8D_UP	−2.27	0	0
SCHUETZ_BREAST_CANCER_DUCTAL_INVASIVE_UP	−2.26	0	0
KIM_LRRC3B_TARGETS	−2.25	0	0
HUANG_DASATINIB_RESISTANCE_UP	−2.24	0	0
REN_ALVEOLAR_RHABDOMYOSARCOMA_DN	−2.23	0	0
PICCALUGA_ANGIOIMMUNOBLASTIC_LYMPHOMA_UP	−2.23	0	0
KOBAYASHI_EGFR_SIGNALING_24HR_UP	−2.23	0	0
TAKEDA_TARGETS_OF_NUP98_HOXA9_FUSION_10D_UP	−2.21	0	0
HELLEBREKERS_SILENCED_DURING_TUMOR_ANGIOGENESIS	−2.2	0	0
TSAI_RESPONSE_TO_RADIATION_THERAPY	−2.2	0	0
FURUKAWA_DUSP6_TARGETS_PCI35_UP	−2.2	0	0
BOWIE_RESPONSE_TO_EXTRACELLULAR_MATRIX	−2.19	0	0
KRASNOSELSKAYA_ILF3_TARGETS_UP	−2.19	0	0
PETROVA_ENDOTHELIUM_LYMPHATIC_VS_BLOOD_DN	−2.19	0	0
ROZANOV_MMP14_TARGETS_UP	−2.18	0	0
CHICAS_RB1_TARGETS_CONFLUENT	−2.18	0	0
FARMER_BREAST_CANCER_CLUSTER_1	−2.16	0	0
AZARE_NEOPLASTIC_TRANSFORMATION_BY_STAT3_DN	−2.15	0	0

(*Continued*)

DiI Bright Enrichment			
**GS**	**NES**	**NOM p-val**	**FDR q-val**
**follow link to MSigDB**			
XU_AKT1_TARGETS_6HR	−2.15	0	0
CUI_TCF21_TARGETS_UP	−2.14	0	0
KEGG_TYPE_I_DIABETES_MELLITUS	−2.12	0	0.001
WU_CELL_MIGRATION	−2.11	0	0.001
ZHU_CMV_ALL_DN	−2.11	0	0.001
NAKAYAMA_SOFT_TISSUE_TUMORS_PCA1_UP	−2.11	0	0.001
LIANG_SILENCED_BY_METHYLATION_2	−2.11	0	0.001
RODWELL_AGING_KIDNEY_NO_BLOOD_UP	−2.1	0	0.001
LIU_PROSTATE_CANCER_DN	−2.09	0	0.001
LANDIS_BREAST_CANCER_PROGRESSION_DN	−2.09	0	0.001
RADAEVA_RESPONSE_TO_IFNA1_UP	−2.09	0	0.001
COLIN_PILOCYTIC_ASTROCYTOMA_VS_GLIOBLASTOMA_UP	−2.08	0	0.001
COWLING_MYCN_TARGETS	−2.08	0	0.001
ROY_WOUND_BLOOD_VESSEL_UP	−2.08	0	0.001
HECKER_IFNB1_TARGETS	−2.07	0	0.001
RUIZ_TNC_TARGETS_UP	−2.07	0	0.001
SANA_TNF_SIGNALING_DN	−2.07	0	0.001
MAHADEVAN_IMATINIB_RESISTANCE_UP	−2.06	0	0.001
LIANG_SILENCED_BY_METHYLATION_UP	−2.05	0	0.002
KANG_GIST_WITH_PDGFRA_UP	−2.05	0	0.001
MISSIAGLIA_REGULATED_BY_METHYLATION_UP	−2.05	0	0.001
MIYAGAWA_TARGETS_OF_EWSR1_ETS_FUSIONS_DN	−2.04	0	0.002
CROMER_TUMORIGENESIS_UP	−2.04	0	0.002

DiI Bright Enrichment			
**GS**	**NES**	**NOM p-val**	**FDR q-val**
**follow link to MSigDB**			
BROWNE_HCMV_INFECTION_24HR_DN	−2.04	0	0.002
JOHNSTONE_PARVB_TARGETS_2_UP	−2.04	0	0.002
KEGG_ALLOGRAFT_REJECTION	−2.04	0	0.002
SANA_RESPONSE_TO_IFNG_UP	−2.03	0	0.002
BOQUEST_STEM_CELL_UP	−2.03	0	0.002
WONG_ENDMETRIUM_CANCER_DN	−2.03	0	0.002
KARLSSON_TGFB1_TARGETS_DN	−2.03	0	0.002
KEGG_GRAFT_VERSUS_HOST_DISEASE	−2.02	0	0.002
SUNG_METASTASIS_STROMA_UP	−2.02	0	0.002
FRIDMAN_IMMORTALIZATION_DN	−2.02	0	0.002
KANNAN_TP53_TARGETS_UP	−2.01	0	0.002
MANALO_HYPOXIA_UP	−2.01	0	0.002
KAAB_HEART_ATRIUM_VS_VENTRICLE_UP	−2.01	0	0.003
WARTERS_RESPONSE_TO_IR_SKIN	−2.01	0	0.003
ZHANG_INTERFERON_RESPONSE	−2.01	0	0.003
LAIHO_COLORECTAL_CANCER_SERRATED_UP	−2	0	0.003
SENESE_HDAC1_TARGETS_DN	−2	0	0.003
HENDRICKS_SMARCA4_TARGETS_UP	−2	0	0.003
BENNETT_SYSTEMIC_LUPUS_ERYTHEMATOSUS	−1.99	0	0.003
LINDSTEDT_DENDRITIC_CELL_MATURATION_D	−1.99	0.002	0.003
KEGG_CELL_ADHESION_MOLECULES_CAMS	−1.99	0	0.003
GILDEA_METASTASIS	−1.99	0	0.003

(*Continued*)

	DiI Bright Enrichment		
**GS**	**NES**	**NOM p-val**	**FDR q-val**
**follow link to MSigDB**			
MCCLUNG_DELTA_FOSB_TARGETS_2WK	−1.99	0	0.003
PID_SYNDECAN_1_PATHWAY	−1.99	0	0.003
WIELAND_UP_BY_HBV_INFECTION	−1.98	0	0.003
THUM_MIR21_TARGETS_HEART_DISEASE_UP	−1.98	0	0.003
BECKER_TAMOXIFEN_RESISTANCE_UP	−1.98	0	0.003
GAUSSMANN_MLL_AF4_FUSION_TARGETS_F_UP	−1.98	0	0.003
ZHAN_MULTIPLE_MYELOMA_LB_DN	−1.97	0	0.003
UROSEVIC_RESPONSE_TO_IMIQUIMOD	−1.97	0	0.003
DER_IFN_ALPHA_RESPONSE_UP	−1.97	0	0.004
KINSEY_TARGETS_OF_EWSR1_FLII_FUSION_DN	−1.97	0	0.004
CROONQUIST_IL6_DEPRIVATION_UP	−1.96	0	0.004
DANG_REGULATED_BY_MYC_DN	−1.96	0	0.004
ZIRN_TRETINOIN_RESPONSE_UP	−1.95	0	0.004
REACTOME_INTERFERON_ALPHA_BETA_SIGNALING	−1.95	0	0.004
HARRIS_BRAIN_CANCER_PROGENITORS	−1.95	0.002	0.004
GUENTHER_GROWTH_SPHERICAL_VS_ADHERENT_DN	−1.95	0	0.004
LIM_MAMMARY_STEM_CELL_UP	−1.95	0	0.004
REACTOME_NCAM1_INTERACTIONS	−1.95	0.002	0.004
EINAV_INTERFERON_SIGNATURE_IN_CANCER	−1.95	0	0.004
VANTVEER_BREAST_CANCER_METASTASIS_UP	−1.95	0	0.004
DASU_IL6_SIGNALING_SCAR_DN	−1.95	0	0.004

DiI Bright Enrichment			
**GS**	**NES**	**NOM p-val**	**FDR q-val**
**follow link to MSigDB**			
BROWNE_INTERFERON_RESPONSIVE_GENES	−1.94	0	0.004
JI_CARCINOGENESIS_BY_KRAS_AND_STK11_DN	−1.94	0.002	0.004
KEGG_VIRAL_MYOCARDITIS	−1.94	0	0.005
TURASHVILI_BREAST_LOBULAR_CARCINOMA_VS_DUCTAL_NORMAL_UP	−1.94	0	0.004
HELLER_SILENCED_BY_METHYLATION_UP	−1.94	0	0.004
PETRETTO_CARDIAC_HYPERTROPHY	−1.93	0	0.005
KORKOLA_YOLK_SAC_TUMOR	−1.93	0	0.005
POTTI_TOPOTECAN_SENSITIVITY	−1.93	0	0.005
NIELSEN_SYNOVIAL_SARCOMA_DN	−1.93	0	0.005
POOLA_INVASIVE_BREAST_CANCER_DN	−1.92	0	0.005
PETROVA_PROX1_TARGETS_DN	−1.92	0	0.005
DASU_IL6_SIGNALING_UP	−1.92	0	0.005
ISSAEVA_MLL2_TARGETS	−1.92	0	0.005
WOTTON_RUNX_TARGETS_UP	−1.92	0.004	0.005
SUZUKI_RESPONSE_TO_TSA_AND_DECITABINE_1A	−1.91	0.002	0.006
CERVERA_SDHB_TARGETS_2	−1.91	0	0.006
CLASPER_LYMPHATIC_VESSELS_DURING_METASTASIS_UP	−1.91	0.002	0.006
XU_HGF_TARGETS_INDUCED_BY_AKT1_6HR	−1.91	0.004	0.006
REACTOME_INTEGRIN_CELL_SURFACE_INTERACTIONS	−1.9	0	0.006
KOYAMA_SEMA3B_TARGETS_UP	−1.9	0	0.006

(*Continued*)

DiI Bright Enrichment			
**GS**	**NES**	**NOM p-val**	**FDR q-val**
**follow link to MSigDB**			
BERENJENO_TRANSFORMED_BY_RHOA_DN	−1.9	0	0.006
GRANDVAUX_IRF3_TARGETS_UP	−1.9	0	0.007
AMUNDSON_GAMMA_RADIATION_RESISTANCE	−1.89	0	0.008
CORRE_MULTIPLE_MYELOMA_UP	−1.89	0	0.008
TURASHVILI_BREAST_LOBULAR_CARCINOMA_VS_LOBULAR_NORMAL_DN	−1.89	0	0.008
KANG_CISPLATIN_RESISTANCE_UP	−1.88	0.002	0.008
GARCIA_TARGETS_OF_FLI1_AND_DAX1_UP	−1.88	0.002	0.008
VERHAAK_AML_WITH_NPM1_MUTATED_DN	−1.88	0	0.008
KEGG_AUTOIMMUNE_THYROID_DISEASE	−1.88	0	0.009
LEE_NEURAL_CREST_STEM_CELL_UP	−1.88	0	0.009
IGLESIAS_E2F_TARGETS_UP	−1.88	0	0.009
MCCABE_HOXC6_TARGETS_DN	−1.87	0	0.009
SMID_BREAST_CANCER_RELAPSE_IN_BRAIN_DN	−1.87	0	0.009
GAURNIER_PSMD4_TARGETS	−1.87	0	0.009
PID_AVB3_INTEGRIN_PATHWAY	−1.86	0	0.01
RIGGI_EWING_SARCOMA_PROGENITOR_DN	−1.86	0	0.01
REACTOME_COLLAGEN_FORMATION	−1.86	0	0.01
REACTOME_HDL_MEDIATED_LIPID_TRANSPORT	−1.86	0.002	0.01
IZADPANAH_STEM_CELL_ADIPOSE_VS_BONE_DN	−1.86	0	0.01
RODWELL_AGING_KIDNEY_UP	−1.86	0	0.01

DiI Bright Enrichment			
**GS**	**NES**	**NOM p-val**	**FDR q-val**
**follow link to MSigDB**			
VALK_AML_CLUSTER_11	−1.86	0.002	0.01
HUANG_FOXA2_TARGETS_DN	−1.85	0	0.011
REACTOME_EXTRACELLULAR_MATRIX_ORGANIZATION	−1.85	0	0.011
KONDO_EZH2_TARGETS	−1.85	0	0.011
TRAYNOR_RETT_SYNDROM_UP	−1.85	0.004	0.011
LINDGREN_BLADDER_CANCER_CLUSTER_2B	−1.85	0	0.011
ACEVEDO_FGFR1_TARGETS_IN_PROSTATE_CANCER_MODEL_DN	−1.85	0	0.012
YANG_BCL3_TARGETS_UP	−1.84	0	0.012
ZHANG_GATA6_TARGETS_DN	−1.84	0	0.012
KEGG_ECM_RECEPTOR_INTERACTION	−1.84	0	0.012
VART_KSHV_INFECTION_ANGIOGENIC_MARKERS_UP	−1.84	0	0.012
DUNNE_TARGETS_OF_AML1_MTG8_FUSION_DN	−1.84	0.002	0.012
CHANG_IMMORTALIZED_BY_HPV31_DN	−1.84	0	0.013
FRIDMAN_SENESCENCE_UP	−1.84	0	0.012
WESTON_VEGFA_TARGETS_12HR	−1.84	0.002	0.013
KERLEY_RESPONSE_TO_CISPLATIN_UP	−1.83	0	0.013
REACTOME_PYRIMIDINE_METABOLISM	−1.83	0	0.013
ICHIBA_GRAFT_VERSUS_HOST_DISEASE_D7_UP	−1.83	0	0.013
PHESSE_TARGETS_OF_APC_AND_MBD2_UP	−1.83	0.002	0.013
BAELDE_DIABETIC_NEPHROPATHY_DN	−1.83	0	0.014
VANASSE_BCL2_TARGETS_UP	−1.82	0.002	0.014

(*Continued*)

DiI Bright Enrichment			
**GS**	**NES**	**NOM p-val**	**FDR q-val**
**follow link to MSigDB**			
PID_INTEGRIN3_PATHWAY	−1.82	0	0.015
FUJII_YBX1_TARGETS_UP	−1.82	0	0.015
ROSS_AML_WITH_PML_RARA_FUSION	−1.82	0	0.015
TUOMISTO_TUMOR_SUPPRESSION_BY_COL13A1_UP	−1.81	0.002	0.016
DELYS_THYROID_CANCER_UP	−1.81	0	0.016
JECHLINGER_EPITHELIAL_TO_MESENCHYMAL_TRANSITION_UP	−1.81	0	0.016
BRUECKNER_TARGETS_OF_MIRLET7A3_DN	−1.81	0	0.017
CHIARADONNA_NEOPLASTIC_TRANSFORMATION_KRAS_DN	−1.81	0	0.017
CASTELLANO_NRAS_TARGETS_UP	−1.81	0	0.017
HAN_SATB1_TARGETS_UP	−1.81	0	0.017
LEIN_CHOROID_PLEXUS_MARKERS	−1.81	0	0.017
ONDER_CDH1_TARGETS_2_UP	−1.8	0	0.017
SASAI_RESISTANCE_TO_NEOPLASTIC_TRANSFROMATION	−1.8	0.002	0.017
NEWMAN_ERCC6_TARGETS_DN	−1.8	0.002	0.017
TSUNODA_CISPLATIN_RESISTANCE_DN	−1.8	0	0.017
LU_AGING_BRAIN_UP	−1.8	0	0.017
BERTUCCI_MEDULLARY_VS_DUCTAL_BREAST_CANCER_DN	−1.8	0	0.018
ZHANG_ANTIVIRAL_RESPONSE_TO_RIBAVIRIN_UP	−1.8	0.004	0.018
FRASOR_RESPONSE_TO_ESTRADIOL_DN	−1.8	0	0.018
NIELSEN_SCHWANNOMA_UP	−1.8	0	0.018

DiI Bright Enrichment			
**GS**	**NES**	**NOM p-val**	**FDR q-val**
**follow link to MSigDB**			
BURTON_ADIPOGENESIS_7	−1.8	0	0.018
ZHANG_TLX_TARGETS_DN	−1.79	0	0.018
DUTERTRE_ESTRADIOL_RESPONSE_24HR_DN	−1.79	0	0.018
DIRMEIER_LMP1_RESPONSE_LATE_DN	−1.79	0.002	0.018
MCLACHLAN_DENTAL_CARIES_UP	−1.79	0	0.019
MIKKELSEN_MEF_LCP_WITH_H3K4ME3	−1.79	0	0.019
DER_IFN_BETA_RESPONSE_UP	−1.79	0	0.018
DEMAGALHAES_AGING_UP	−1.79	0	0.019
GRADE_COLON_AND_RECTAL_CANCER_DN	−1.79	0	0.019
HELLER_HDAC_TARGETS_SILENCED_BY_METHYLATION_UP	−1.79	0	0.019
LOPEZ_MESOTHELIOMA_SURVIVAL_OVERALL_DN	−1.79	0.002	0.019
JOHNSTONE_PARVB_TARGETS_3_UP	−1.79	0	0.019
ONDER_CDH1_TARGETS_1_UP	−1.79	0	0.019
PEDERSEN_METASTASIS_BY_ERBB2_ISOFORM_4	−1.79	0	0.019
LEE_LIVER_CANCER_DENA_UP	−1.78	0.002	0.019
BOQUEST_STEM_CELL_CULTURED_VS_FRESH_UP	−1.78	0	0.019
HOELZEL_NF1_TARGETS_UP	−1.78	0	0.02
SENGUPTA_NASOPHARYNGEAL_CARCINOMA_WITH_LMP1_DN	−1.78	0	0.02
WINZEN_DEGRADED_VIA_KHSRP	−1.78	0	0.02
SATO_SILENCED_BY_METHYLATION_IN_PANCREATIC_CANCER_1	−1.78	0	0.02

(*Continued*)

DiI Bright Enrichment			
**GS**	**NES**	**NOM p-val**	**FDR q-val**
**follow link to MSigDB**			
ELVIDGE_HIF1A_AND_HIF2A_TARGETS_DN	−1.78	0	0.02
FARMER_BREAST_CANCER_CLUSTER_5	−1.78	0.006	0.02
WAMUNYOKOLI_OVARIAN_CANCER_LMP_DN	−1.77	0	0.021
CHANG_CORE_SERUM_RESPONSE_DN	−1.77	0	0.021
TAKEDA_TARGETS_OF_NUP98_HOXA9_FUSION_16D_UP	−1.77	0	0.021
CERVERA_SDHB_TARGETS_1_UP	−1.77	0	0.022
SANA_TNF_SIGNALING_UP	−1.77	0.002	0.022
PLASARI_TGFB1_TARGETS_10HR_DN	−1.77	0	0.022
KEGG_GLYCOSAMINOGLYCAN_BIOSYNTHESIS_CHONDROITIN_SULFATE	−1.77	0.002	0.022
PEREZ_TP63_TARGETS	−1.77	0	0.022
TONKS_TARGETS_OF_RUNX1_RUNX1T1_FUSION_HSC_UP	−1.77	0	0.022
CHANDRAN_METASTASIS_DN	−1.77	0	0.022
VERRECCHIA_EARLY_RESPONSE_TO_TGFB1	−1.77	0	0.022
ROZANOV_MMP14_TARGETS_SUBSET	−1.76	0.004	0.022
SHARMA_PILOCYTIC_ASTROCYTOMA_LOCATION_UP	−1.76	0.004	0.022
EBAUER_TARGETS_OF_PAX3_FOXO1_FUSION_UP	−1.76	0	0.022
SMID_BREAST_CANCER_LUMINAL_B_UP	−1.76	0	0.022
LI_WILMS_TUMOR_VS_FETAL_KIDNEY_1_UP	−1.76	0	0.022
RIGGINS_TAMOXIFEN_RESISTANCE_DN	−1.76	0	0.023

DiI Bright Enrichment			
GS	NES	NOM p-val	FDR q-val
follow link to MSigDB			
ZEMBUTSU_SENSITIVITY_TO_VINBLASTINE	−1.76	0	0.023
DUNNE_TARGETS_OF_AML1_MTG8_FUSION_UP	−1.76	0	0.023
PEREZ_TP53_AND_TP63_TARGETS	−1.75	0	0.024
KOKKINAKIS_METHIONINE_DEPRIVATION_96HR_UP	−1.75	0	0.024
ZHAN_V1_LATE_DIFFERENTIATION_GENES_UP	−1.75	0.004	0.024
PASINI_SUZ12_TARGETS_DN	−1.75	0	0.024
KEGG_ASTHMA	−1.75	0.004	0.024
ICHIBA_GRAFT_VERSUS_HOST_DISEASE_35D_UP	−1.75	0	0.025
BOYAULT_LIVER_CANCER_SUBCLASS_G5_DN	−1.75	0.004	0.025
KIM_WT1_TARGETS_12HR_UP	−1.75	0	0.025
LIU_SMARCA4_TARGETS	−1.75	0.002	0.025
REACTOME_INTERFERON_SIGNALING	−1.75	0	0.026
PAPASPYRIDONOS_UNSTABLE_ATEROSCLEROTIC_PLAQUE_DN	−1.74	0.002	0.026
CHIANG_LIVER_CANCER_SUBCLASS_INTERFERON_UP	−1.74	0.009	0.026
LABBE_TGFB1_TARGETS_UP	−1.74	0	0.026
URS_ADIPOCYTE_DIFFERENTIATION_DN	−1.74	0.005	0.026
MCLACHLAN_DENTAL_CARIES_DN	−1.74	0	0.026
YAO_TEMPORAL_RESPONSE_TO_PROGESTERONE_CLUSTER_16	−1.74	0.003	0.028

(Continued)

DiI Bright Enrichment			
GS	NES	NOM p-val	FDR q-val
follow link to MSigDB			
DOANE_BREAST_CANCER_ESR1_UP	−1.74	0.002	0.028
VALK_AML_WITH_EVI1	−1.74	0.009	0.027
LEIN_NEURON_MARKERS	−1.73	0.002	0.028
HELLER_HDAC_TARGETS_UP	−1.73	0	0.028
VALK_AML_WITH_FLT3_ITD	−1.73	0.002	0.028
CHEMNITZ_RESPONSE_TO_PROSTAGLANDIN_E2_DN	−1.73	0	0.029
KEGG_REGULATION_OF_ACTIN_CYTOSKELETON	−1.73	0	0.029
REACTOME_NEGATIVE_REGULATORS_OF_RIG_I_MDA5_SIGNALING	−1.73	0	0.029
SERVITJA_ISLET_HNF1A_TARGETS_UP	−1.73	0	0.029
MARKEY_RB1_ACUTE_LOF_UP	−1.72	0	0.03
TONKS_TARGETS_OF_RUNX1_RUNX1T1_FUSION_ERYTHROCYTE_UP	−1.72	0	0.03
CHARAFE_BREAST_CANCER_LUMINAL_VS_BASAL_DN	−1.72	0	0.03
CHARAFE_BREAST_CANCER_LUMINAL_VS_MESENCHYMAL_DN	−1.72	0	0.03
ELVIDGE_HYPOXIA_UP	−1.72	0	0.03
IVANOVA_HEMATOPOIESIS_STEM_CELL_LONG_TERM	−1.72	0	0.03
GRUETZMANN_PANCREATIC_CANCER_UP	−1.72	0	0.03
SMID_BREAST_CANCER_LUMINAL_B_DN	−1.72	0	0.03
HIRSCH_CELLULAR_TRANSFORMATION_SIGNATURE_DN	−1.72	0	0.03
GARGALOVIC_RESPONSE_TO_OXIDIZED_PHOSPHOLIPIDS_GREY_DN	−1.72	0.002	0.03

DiI Bright Enrichment			
**GS**	**NES**	**NOM p-val**	**FDR q-val**
**follow link to MSigDB**			
HEIDENBLAD_AMPLICON_8Q24_DN	−1.72	0.002	0.031
REACTOME_GLYCOSAMINOGLYCAN_METABOLISM	−1.72	0.002	0.031
LIEN_BREAST_CARCINOMA_METAPLASTIC	−1.72	0.002	0.031
NADERI_BREAST_CANCER_PROGNOSIS_DN	−1.72	0.006	0.031
ELVIDGE_HIF1A_TARGETS_DN	−1.71	0.002	0.031
REACTOME_COMPLEMENT_CASCADE	−1.71	0.007	0.031
REACTOME_SIGNALING_BY_PDGF	−1.71	0.002	0.032
HORIUCHI_WTAP_TARGETS_UP	−1.71	0	0.032
YAO_HOXA10_TARGETS_VIA_PROGESTERONE_DN	−1.71	0.004	0.033
BROWNE_HCMV_INFECTION_4HR_UP	−1.71	0.002	0.033
DER_IFN_GAMMA_RESPONSE_UP	−1.71	0	0.033
ALONSO_METASTASIS_EMT_UP	−1.71	0.005	0.033
SAMOLS_TARGETS_OF_KHSV_MIRNAS_DN	−1.71	0.002	0.033
MASRI_RESISTANCE_TO_TAMOXIFEN_AND_AROMATASE_INHIBITORS_DN	−1.7	0.025	0.034
DURAND_STROMA_MAX_UP	−1.7	0	0.034
LANDIS_ERBB2_BREAST_TUMORS_324_DN	−1.7	0.002	0.034
CAIRO_LIVER_DEVELOPMENT_DN	−1.7	0	0.034
WESTON_VEGFA_TARGETS_6HR	−1.7	0.005	0.034
REACTOME_INTERFERON_GAMMA_SIGNALING	−1.7	0	0.034

(*Continued*)

DiI Bright Enrichment			
GS	NES	NOM p-val	FDR q-val
follow link to MSigDB			
CROONQUIST_STROMAL_STIMULATION_UP	−1.7	0.004	0.035
BOQUEST_STEM_CELL_DN	−1.7	0	0.035
BIOCARTA_RHO_PATHWAY	−1.69	0.009	0.036
VALK_AML_CLUSTER_10	−1.69	0.009	0.037
YAO_TEMPORAL_RESPONSE_TO_PROGESTERONE_CLUSTER_15	−1.69	0.005	0.037
VALK_AML_CLUSTER_4	−1.69	0.005	0.037
HANN_RESISTANCE_TO_BCL2_INHIBITOR_UP	−1.69	0.007	0.037
SCHAEFFER_PROSTATE_DEVELOPMENT_48HR_DN	−1.69	0	0.038
HAN_SATB1_TARGETS_DN	−1.69	0	0.038
GAUSSMANN_MLL_AF4_FUSION_TARGETS_E_UP	−1.69	0.002	0.038
VALK_AML_CLUSTER_13	−1.69	0.006	0.038
VALK_AML_CLUSTER_12	−1.69	0.004	0.038
VECCHI_GASTRIC_CANCER_EARLY_DN	−1.69	0	0.038
PID_INTEGRIN_CS_PATHWAY	−1.69	0.013	0.038
HOSHIDA_LIVER_CANCER_SUBCLASS_S1	−1.68	0	0.038
VECCHI_GASTRIC_CANCER_ADVANCED_VS_EARLY_UP	−1.68	0.002	0.038
WELCSH_BRCA1_TARGETS_UP	−1.68	0	0.04
VERHAAK_GLIOBLASTOMA_NEURAL	−1.68	0	0.04
ZHANG_TLX_TARGETS_60HR_UP	−1.68	0	0.041
THUM_SYSTOLIC_HEART_FAILURE_UP	−1.68	0	0.041
RICKMAN_TUMOR_DIFFERENTIATED_MODERATELY_VS_POORLY_UP	−1.67	0.002	0.041

DiI Bright Enrichment			
**GS**	**NES**	**NOM p-val**	**FDR q-val**
**follow link to MSigDB**			
GREENBAUM_E2A_TARGETS_DN	−1.67	0.013	0.041
KEGG_INTESTINAL_IMMUNE_NETWORK_FOR_IGA_PRODUCTION	−1.67	0.002	0.041
ELVIDGE_HYPOXIA_BY_DMOG_UP	−1.67	0.002	0.041
KEGG_FOCAL_ADHESION	−1.67	0	0.041
CHEBOTAEV_GR_TARGETS_DN	−1.67	0	0.042
YAMASHITA_METHYLATED_IN_PROSTATE_CANCER	−1.67	0.004	0.042
NAKAMURA_ADIPOGENESIS_LATE_DN	−1.67	0.002	0.042
HUPER_BREAST_BASAL_VS_LUMINAL_DN	−1.67	0.003	0.044
ACEVEDO_LIVER_CANCER_WITH_H3K27ME3_UP	−1.66	0	0.044
SMID_BREAST_CANCER_NORMAL_LIKE_UP	−1.66	0	0.045
ZHENG_IL22_SIGNALING_UP	−1.66	0.01	0.045
MAHADEVAN_GIST_MORPHOLOGICAL_SWITCH	−1.66	0.017	0.045
REACTOME_KERATAN_SULFATE_KERATIN_METABOLISM	−1.66	0.013	0.045
JOHANSSON_BRAIN_CANCER_EARLY_VS_LATE_DN	−1.66	0.007	0.045
SCIBETTA_KDM5B_TARGETS_DN	−1.66	0.007	0.046
REICHERT_MITOSIS_LIN9_TARGETS	−1.66	0.022	0.046
MCMURRAY_TP53_HRAS_COOPERATION_RESPONSE_DN	−1.66	0.002	0.046
MIKKELSEN_MCV6_LCP_WITH_H3K4ME3	−1.66	0	0.046

(*Continued*)

DiI Bright Enrichment			
**GS**	**NES**	**NOM p-val**	**FDR q-val**
**follow link to MSigDB**			
LINDVALL_IMMORTALIZED_BY_TERT_DN	−1.66	0.002	0.046
CLASPER_LYMPHATIC_VESSELS_DURING_METASTASIS_DN	−1.66	0.009	0.045
PID_LYMPHANGIOGENESIS_PATHWAY	−1.66	0.007	0.046
LEE_AGING_NEOCORTEX_DN	−1.66	0	0.046
POMEROY_MEDULLOBLASTOMA_DESMOPLASIC_VS_CLASSIC_DN	−1.65	0.007	0.048
LA_MEN1_TARGETS	−1.65	0.013	0.048
TARTE_PLASMA_CELL_VS_PLASMABLAST_UP	−1.65	0	0.049
**DiI Dull Enrichment**			
**GS**	**NES**	**NOM p-val**	**FDR q-val**
**follow link to MSigDB**			
MANALO_HYPOXIA_DN	2.23	0	0.003
REACTOME_RNA_POL_II_TRANSCRIPTION	2.14	0	0.01
REACTOME_PROCESSING_OF_CAPPED_INTRON_CONTAINING_PRE_MRNA	2.12	0	0.009
REACTOME_MRNA_SPLICING	2.1	0	0.01
SCHLOSSER_MYC_TARGETS_AND_SERUM_RESPONSE_DN	2.09	0	0.009
REACTOME_MRNA_PROCESSING	2.08	0	0.009
Genes up-regulated in epithelial-like melanoma cell line vs mesenchymal-like cell lines (Supplementary Table S1, current study)	2.07	0	0.001
WALLACE_JAK2_TARGETS_UP	2.03	0	0.017

DiI Bright Enrichment			
**GS**	**NES**	**NOM p-val**	**FDR q-val**
**follow link to MSigDB**			
REACTOME_METABOLISM_OF_NON_CODING_RNA	2.03	0	0.015
REACTOME_ELONGATION_ARREST_AND_RECOVERY	2	0	0.022
PICCALUGA_ANGIOIMMUNOBLASTIC_LYMPHOMA_DN	1.99	0	0.021
PID_MAPKTRKPATHWAY	1.98	0	0.024
SCHUHMACHER_MYC_TARGETS_UP	1.96	0	0.027
KEGG_SPLICEOSOME	1.95	0	0.029
REACTOME_HIV_LIFE_CYCLE	1.94	0	0.031
MMS_MOUSE_LYMPH_HIGH_4HRS_UP	1.93	0	0.034
REACTOME_LATE_PHASE_OF_HIV_LIFE_CYCLE	1.93	0	0.032
REACTOME_RNA_POL_III_TRANSCRIPTION	1.93	0	0.03
GARCIA_TARGETS_OF_FLI1_AND_DAX1_DN	1.9	0	0.042
MUELLER_PLURINET	1.9	0	0.04
REACTOME_FORMATION_OF_RNA_POL_II_ELONGATION_COMPLEX_	1.9	0.002	0.039
PID_ATM_PATHWAY	1.89	0.002	0.042
PID_ATF2_PATHWAY	1.88	0	0.041
REACTOME_MRNA_SPLICING_MINOR_PATHWAY	1.88	0	0.041
